# SH2B3 Promotes PD-L1-Mediated Immune Evasion in Glioblastoma via BRCC3-Dependent IGF2BP2 Stabilization

**DOI:** 10.7150/ijbs.134558

**Published:** 2026-08-24

**Authors:** Rongqing Li, Jiahui Wang, Xiangrui Meng, Jiahua Pan, Xin Chen, Xin Pan, Wei Li, Shigang Qiao, Li Qian

**Affiliations:** 1 Key Laboratory of the Jiangsu Higher Education Institutions for Nucleic Acid & Cell Fate Regulation (Yangzhou University), School of Basic Medical Sciences & School of Public Health, Faculty of Medicine, Yangzhou University, Yangzhou, 225009, China.; 2 Department of Respiratory Medicine, Taizhou Second People's Hospital Affiliated to Yangzhou University, Yangzhou University, Yangzhou, 225001, China.; 3 Department of Clinical Laboratory, Affiliated Jinhua Hospital, Zhejiang University School of Medicine, Jinhua, 321000, China.; 4 Department of Cardiology, Central Laboratory, Institute of Cardiovascular Disease, Yangzhou Key Lab of Innovation Frontiers in Cardiovascular Disease, Affiliated Hospital of Yangzhou University, Yangzhou University, Yangzhou, Jiangsu, 225001, China.; 5 Department of Anesthesiology/Department Center Laboratory, Kunshan Hospital of Chinese Medicine, Affiliated Hospital of Yangzhou University, Kunshan, 215300, Jiangsu, China.; 6 Jiangsu Key Laboratory of Zoonosis, Yangzhou University, Yangzhou, 225009, China.

**Keywords:** SH2B3, BRCC3, IGF2BP2, PD-L1, glioblastoma

## Abstract

Glioblastoma (GBM) is characterized by aggressive progression and profound immune evasion, yet the underlying mechanisms remain incompletely understood. Here, we identify the adaptor protein SH2B3 as a critical driver of immune evasion in glioblastoma and show that elevated SH2B3 expression is associated with higher glioma grade and poorer survival in public datasets and clinical data. Although SH2B3 modulation did not significantly affect GBM cell proliferation, migration, or invasion *in vitro* assays, it significantly altered tumorigenesis *in vivo*, with overexpression enhancing and knockdown suppressing tumor growth, suggesting that microenvironmental factors may contribute to the *in vivo* phenotype. We further show that SH2B3 promotes immune evasion at least in part by upregulating PD-L1 expression. Mechanistically, SH2B3 facilitates BRCC3-dependent removal of K63-linked polyubiquitin chains from the m⁶A reader IGF2BP2, thereby stabilizing IGF2BP2 and preventing its autophagy-lysosome-dependent degradation. The stabilized IGF2BP2 then enhances the stability and translation of m⁶A-marked PD-L1 transcripts through m⁶A-dependent transcript recognition. Collectively, our findings reveal a novel SH2B3/BRCC3/IGF2BP2 axis connecting protein deubiquitination and RNA epigenetics to PD-L1-mediated immune evasion, highlighting SH2B3 as a promising therapeutic target for glioblastoma immunotherapy.

## Introduction

Glioblastoma (GBM) is the most common and aggressive malignant primary brain tumor in adults 1. Its clinical course is marked by rapid recurrence, profound therapeutic resistance, and an exceptionally poor prognosis, with a median overall survival of less than 15 months despite current standard-of-care treatments 2. This therapeutic deadlock largely reflects a multifactorial resistance landscape characterized by extensive intratumoral heterogeneity, a profoundly immunosuppressive tumor microenvironment (TME), and restricted drug delivery imposed by the blood-brain barrier (BBB) 3, 4. Consequently, the development of innovative and more effective therapeutic strategies remains an urgent clinical priority. Recent progress in immunotherapeutic approaches, including immune checkpoint blockade, chimeric antigen receptor (CAR) T-cell therapy, cancer vaccines, and oncolytic virotherapy, has begun reshaping the GBM treatment paradigm 5, 6.

A central obstacle in GBM management is its sophisticated immune evasion machinery 7. Programmed death-ligand 1 (PD-L1, encoded by the *CD274* gene), as one of the most critical immune checkpoints, binds to PD-1 on T cells to mediate T-cell exhaustion, ultimately enabling immune evasion 8. Within the TME, both tumor cells and tumor-associated antigen-presenting cells (APCs) exhibit high PD-L1 expression, whereas persistent antigen exposure drives tumor-infiltrating lymphocytes (TILs) to overexpress PD-1 8-10. PD-L1/PD-1 engagement induces T-cell apoptosis, anergy, and exhaustion, thereby suppressing the activation, proliferation, and antitumor functionality of tumor antigen-specific CD8⁺ T cells to facilitate immune evasion 11. Consequently, targeting tumor-associated PD-L1 may serve as a potential therapeutic direction for glioblastoma 12-14, and clinical studies of nivolumab, pembrolizumab, and atezolizumab have observed modest anti-tumor responses in selected GBM patient populations 12-15. However, their clinical efficacy remains limited, largely due to BBB constraints, a highly suppressive immune milieu, and marked tumor heterogeneity 16. This underscores the need for both innovative PD-L1 inhibitors and comprehensive investigation into endogenous PD-L1 regulatory mechanisms. A broad spectrum of epigenetic remodeling events, including aberrant DNA methylation, histone modifications and epitranscriptomic alterations, can jointly orchestrate transcriptional and post-transcriptional PD-L1 reprogramming in glioma cells, serving as important contributors to tumor immune evasion 9, 17. Such evidence highlights epigenetic modulation as a promising avenue for PD-L1-targeted therapy.

GBM development is accompanied by extensive dysregulation of epigenetic processes, including DNA methylation, histone acetylation, microRNA-mediated gene expression interference 18, and N6-methyladenosine (m⁶A) RNA modification 19. m⁶A, the most abundant internal RNA modification, is dynamically controlled by writer, eraser, and reader proteins 19. Among these regulators, IGF2BP2 functions as a critical m⁶A “reader” that stabilizes target transcripts in an m⁶A-dependent manner 20. Structurally, IGF2BP2 contains two RNA recognition motifs (RRM1 and RRM2) and four K-homology (KH1-KH4) domains, enabling high-affinity binding to m⁶A-modified mRNAs 21. Within GBM tissues, the expression of the immune checkpoint PD-L1 shows significant correlation with m⁶A methylation regulators 22. The m⁶A modification of PD-L1 mRNA and its impact on immune evasion have been investigated in several tumor types. For instance, the METTL3/IGF2BP3 axis enhances m⁶A modification of PD-L1 mRNA to suppress immune surveillance in breast cancer 23, and ALKBH5 promotes PD-L1-mediated immune evasion through m⁶A-dependent regulation of ZDHHC3 in glioma 24. However, the specific contribution of IGF2BP2 to the recognition and stabilization of m⁶A-marked PD-L1 transcripts remains unclear, particularly in GBM. Notably, multiple m⁶A enrichment sites, hallmarks of m⁶A methylation, have been identified within mature PD-L1 mRNA 25, supporting the relevance of m⁶A-dependent PD-L1 regulation. These observations collectively suggest that targeting PD-L1-mediated immune evasion through modulation of m⁶A modifications represents a promising therapeutic strategy for GBM.

Alongside epigenetic reprogramming, GBM progression also involves extensive post-translational alterations that reshape the stability and function of key oncogenic proteins, including aberrant activity within the ubiquitin-proteasome system and the autophagy-lysosome pathway 26. Among post-translational modifications, ubiquitination acts as a prominent, evolutionarily conserved internal modification in eukaryotes 27. This process entails the covalent attachment of ubiquitin to substrate proteins through a coordinated enzymatic cascade involving E1 ubiquitin-activating enzymes, E2 ubiquitin-conjugating enzymes, and E3 ubiquitin ligases 28. Notably, certain E3 ubiquitin ligases catalyze formation of K63-linked polyubiquitin chains, a modification that does not directly target substrates for proteasomal degradation but rather serves as a critical signal for their clearance via selective autophagy 29. BRCA1/BRCA2-containing complex subunit 3 (BRCC3), a key deubiquitinating enzyme responsible for protein deubiquitination and homeostasis regulation, participates in diverse cellular functions 30. BRCC3-mediated K63-specific deubiquitination of NLRP3 promotes inflammasome activation, whereas CD82 counteracts this effect by directly binding to BRCC3, which consequently enhances NLRP3 ubiquitination and degradation, thereby attenuating the inflammatory response in colitis 31. Beyond its role in NLRP3 inflammasome regulation, BRCC3 has also been implicated in epigenetic control. For instance, BRCC3 together with USP14 deubiquitinates KDM4D at K63-linked chains, preventing its OPTN-mediated autophagic degradation and sustaining H3K9me3 demethylation to promote inflammatory gene transcription 32. In parallel, other deubiquitinases have been shown to regulate the stability of m⁶A demethylases in GBM. Specifically, in a distinct substrate context, USP14 stabilizes ALKBH5 by removing K48-linked ubiquitin chains in an MST4-phosphorylation-dependent manner, thereby sustaining GSC stemness and radioresistance 33; likewise, USP36 directly deubiquitinates and stabilizes ALKBH5, promoting glioblastoma tumorigenesis and chemoresistance 34. However, the role of BRCC3 in regulating IGF2BP2 ubiquitination remains underexplored, particularly in glioblastoma. Collectively, these observations suggest that targeting the stability of epigenetic modifiers through modulation of post-translational modifications represents a promising therapeutic strategy against glioblastoma progression.

The SH2B adaptor protein 3 (SH2B3), also known as LNK, orchestrates signaling cascades downstream of growth factor and cytokine receptors 35. Accumulating evidence indicates that SH2B3 plays context-dependent roles in oncogenesis and immune regulation. For instance, SH2B3 suppresses CD8⁺ T cell cytotoxicity in the tumor microenvironment by inhibiting IFN-STAT1 signaling in melanoma cells 36, and SH2B3 overexpression promotes tumor growth but suppresses migration in ovarian cancer through sustained activation of mitotic signaling pathways 37. In parallel, our group has continuously investigated the biological functions of SH2B3 in tumor immunity and neurological disorders. We recently demonstrated that SH2B3 deficiency attenuates the immunosuppressive capacity of myeloid-derived suppressor cells (MDSCs) via ferroptosis, thereby suppressing tumor development 38. We also identified a SH2B3/CBL/HNRNPA2B1/GPX4 signaling axis that mediates dopaminergic neuron vulnerability to ferroptosis in Parkinson's disease 39. Meanwhile, we noted that SH2B3 is highly expressed in glioma in the TCGA and CGGA databases and is negatively associated with patient prognosis, but its role in glioma and the underlying molecular mechanisms remain to be further investigated.

Consequently, we employed SH2B3-knockdown and overexpression cellular models, together with subcutaneous and orthotopic syngeneic mouse models, to elucidate the role of SH2B3 in glioblastoma progression. Specifically, we investigated whether SH2B3 facilitates BRCC3-dependent removal of K63-linked ubiquitin chains from IGF2BP2, thereby limiting its autophagy-lysosomal degradation, enhancing PD-L1 mRNA stability and promoting glioblastoma immune evasion. These findings provide insights that may facilitate the identification of potential clinically relevant markers, help to clarify the mechanistic basis of immune evasion during glioblastoma progression, and lay the groundwork for the clinical translation of targeted therapies.

## Materials and Methods

### Database analysis

SH2B3 expression in glioma was analyzed using transcriptomic and clinical data from The Cancer Genome Atlas (TCGA; https://cancergenome.nih.gov/) and the Chinese Glioma Genome Atlas (CGGA; http://www.cgga.org.cn/). SH2B3 expression in TCGA low-grade glioma (LGG) and glioblastoma (GBM) samples was compared with that in Genotype-Tissue Expression (GTEx) normal brain samples. Kaplan-Meier survival analyses were subsequently performed to assess the association between SH2B3 expression and patient prognosis.

### Human glioma tissues

Glioma tissues and peritumoral tissue (n = 39) were obtained from the Biobank of Jinhua Central Hospital. Written informed consent was acquired from all patients, and their relevant clinical characteristics are summarized in Table 1. Immediately after resection, specimens were snap-frozen in liquid nitrogen and stored at -80°C. This study was approved by the Jinhua Central Hospital Institutional Ethics Committee (20241920101). All procedures involving human participants were performed in accordance with the ethical standards of the institutional and/or national research committee and with the 1964 Helsinki Declaration and its later amendments or comparable ethical standards.

### Cell culture and reagents

Normal human astrocytes (HA) and the GBM cell lines U87, U251, T98G, A172, and LN229 were obtained from ATCC (American Type Culture Collection). Mouse GBM cell line GL261 was purchased from German Collection of Microorganisms and Cell Cultures (DSMZ). All human GBM cell lines were authenticated by short tandem repeat (STR) analysis. The murine cell line GL261 was authenticated by species-specific PCR and murine STR marker analysis to confirm its murine origin and exclude interspecies cross-contamination. All cell lines were routinely tested and confirmed negative for Mycoplasma contamination using a PCR-based detection kit. All cells were cultured in DMEM (Gibco, USA) supplemented with 10% FBS and 100 U/mL penicillin-streptomycin, and maintained at 37 °C in a humidified 5% CO₂ incubator. MG132 (HY-13259; MCE, USA), chloroquine (CQ; HY-17589A; MCE, USA), 3-methyladenine (3-MA; HY-19312; MCE, USA), bafilomycin A1 (BafA1; HY-100558; MCE, USA), Earle's balanced salt solution (EBSS; H2020; Solarbio, China), anti-PD-L1 antibody (BE0101; Bio X Cell, USA), and etoposide phosphate (HY-13630; MCE, USA) were used as indicated. All drugs were dissolved in DMSO (GC203002, Servicebio, China), PBS, or H₂O, following the manufacturer's instructions.

### Cell Counting Kit-8 (CCK-8) assay

Cell viability was measured with the CCK-8 assay (Abbkine, China). U251, T98G, and GL261 cells were plated in 96-well plates at 3,000 cells/well. After incubation at 37 °C for 0, 12, 24, 48, or 72 hours, 10 μL of CCK-8 reagent was added to each well and incubated for 2 hours under the same conditions. Absorbance at 450 nm was measured on a multimode microplate reader (Thermo Fisher Scientific, USA).

### 5-Ethynyl-2ʹ-deoxyuridine (EdU) proliferation assay

Cells were seeded in 96-well plates at 3,000 cells per well and cultured for 24 hours at 37 °C. After incubation with 10 μM EdU solution (Abbkine, China) for 2 hours, cells were fixed with 4% paraformaldehyde (PFA) and permeabilized with 0.5% Triton X-100 in PBS for 10 minutes. Subsequently, cells were stained with EdU reaction cocktail for 30 minutes at room temperature protected from light, followed by nuclear counterstaining with DAPI. Fluorescence images were acquired on an Olympus microscope (Tokyo, Japan). Proliferating cells were identified by red/green fluorescence, while all nuclei appeared blue. The proliferation rate (%) was calculated as (EdU-positive cells / DAPI-positive cells) × 100. Quantitative analysis of total and proliferating cells was performed using ImageJ software.

### Transwell invasion assay

Matrigel was thawed overnight at 4 °C and diluted with serum-free medium at a ratio of 1:8. The upper chambers of 24-well Transwell inserts (8-μm pore size) were coated with 100 μL of the diluted Matrigel and incubated at 37 °C for 2-4 h to allow polymerization. After gelation, the remaining liquid was gently aspirated, and the coated membranes were rehydrated with 100 μL of serum-free medium at 37 °C for 30 min.

Cells were serum-starved for 12-24 h, harvested, washed twice with PBS, and resuspended in serum-free medium at a density of 5 × 10⁵ cells/mL. A 200-μL aliquot of the cell suspension (1 × 10⁵ cells) was added to the upper chamber. The lower chamber was filled with 600 μL of complete medium containing 10%-20% FBS as a chemoattractant. After incubation at 37 °C for 24 h, the inserts were removed, and non-invading cells on the upper surface were gently wiped off with a cotton swab. The inserts were then washed with PBS, fixed with 4% paraformaldehyde for 30 min, and stained with 0.1% crystal violet for 30 min. After washing with PBS and air-drying, stained cells were observed under a light microscope, and five random fields were selected for counting. The number of invaded cells was normalized to that of the control group and expressed as relative number of cells. Each experiment was performed in at least three independent replicates.

### Wound healing assay

Cells were seeded in 6-well plates at a density of approximately 5 × 10⁵ cells per well and cultured overnight to reach 100% confluence. A straight scratch was made through the cell monolayer using a sterile 200-μL pipette tip. The detached cells were removed by washing gently three times with PBS. The remaining adherent cells were cultured in serum-free medium to minimize the effect of cell proliferation. Images of the scratch were captured at 0 h and 24 h after scratching under a light microscope. The wound area was measured using ImageJ software, and the data were normalized to the control group and expressed as relative wound closure. Each experiment was performed in at least three independent replicates.

### Western blotting

RIPA lysis buffer containing protease inhibitors (Servicebio and Beyotime, China) was used to extract total protein. Protein concentration was measured using a BCA assay kit (Thermo Fisher Scientific, USA). Equal amounts of protein were separated by SDS-polyacrylamide gel electrophoresis (SDS-PAGE) and transferred onto polyvinylidene difluoride (PVDF) membranes (Millipore, USA). After blocking with 5% non-fat milk, membranes were incubated with primary antibodies at 4 °C overnight, followed by incubation with horseradish peroxidase (HRP)-conjugated secondary antibodies (E-AB-1001 and E-AB-1003, Elabscience, China) for 1 h at room temperature. Chemiluminescent signals were detected on a ChemiDoc imaging system (Bio-Rad, USA) using ECL substrate (Abbkine, China). Band densities were quantified using ImageLab software (Bio-Rad) and normalized to β-actin. Primary antibodies used: Anti-SH2B3 (sc-393709, Santa Cruz Biotechnology, USA, 1:500), Anti-PD-L1 (66248-1-Ig, Proteintech, China, 1:2000), Anti-METTL3 (15073-1-AP, Proteintech, China, 1:5000), Anti-METTL14 (26158-1-AP, Proteintech, China, 1:2000), Anti-WTAP (60188-1-Ig, Proteintech, China, 1:5000), Anti-FTO (27226-1-AP, Proteintech, China, 1:1000), Anti-ALKBH5 (16837-1-AP, Proteintech, China, 1:5000), Anti-IGF2BP1 (22803-1-AP, Proteintech, China, 1:5000), Anti-IGF2BP2 (11601-1-AP, Proteintech, China, 1:2000), Anti-IGF2BP3 (14642-1-AP, Proteintech, China, 1:5000), Anti-HNRNPA2/B1 (14813-1-AP, Proteintech, China, 1:5000), Anti-HNRNPC (11760-1-AP, Proteintech, China, 1:5000), Anti-HNRNPG (ER63697, Huabio, China, 1:1000), Anti-YTHDC1 (14392-1-AP, Proteintech, China, 1:1000), Anti-YTHDC2 (27779-1-AP, Proteintech, China, 1:1000), Anti-YTHDF1 (17479-1-AP, Proteintech, China, 1:2000), Anti-YTHDF2 (24744-1-AP, Proteintech, China, 1:2000), Anti-YTHDF3 (25537-1-AP, Proteintech, China, 1:5000), Anti-LAMP2 (66301-1-Ig, Proteintech, China, 1:1000), Anti-BRCC3 (15391-1-AP, Proteintech, China, 1:1000), Anti-Ubiquitin (P23347, Promab, China, 1:500), Anti-K48-linked ubiquitin (CY5964, Abways, China, 1:1000), Anti-K63-linked ubiquitin (CY6579, Abways, China, 1:1000), Anti-β-actin (66009-1-Ig, Proteintech, China, 1:5000).

### Quantitative reverse transcription PCR (RT-qPCR)

Cells or tumor tissues were processed for total RNA extraction using the RNA-easy Isolation Reagent (R701-01, Vazyme, China). HiScript II Q RT SuperMix (+gDNA wiper) (R223, Vazyme, China) was used to reverse-transcribe equal amounts of RNA into cDNA. RT-qPCR was performed using ChamQ Universal SYBR qPCR Master Mix (Q711, Vazyme, China) and gene-specific primers, following the manufacturer's protocol. The mRNA expression levels were calculated by the 2^-ΔΔCt^ method and normalized to β-actin. The primers used are listed below: SH2B3 forward, 5'-TTGAGATGCCTGACAACCTTTAC-3'; SH2B3 reverse, 5'-GCTCTAGGGCTGAGGGAATATG-3'; PD-L1 forward, 5'-TGGCATTTGCTGAACGCATTT-3'; PD-L1 reverse, 5'-TGCAGCCAGGTCTAATTGTTTT-3'; CD24 forward, 5'-CTCCTACCCACGCAGATTTATTC-3'; CD24 reverse, 5'-AGAGTGAGACCACGAAGAGAC-3'; CD47 forward, 5'-AGAAGGTGAAACGATCATCGAGC-3'; CD47 reverse, 5'- CTCATCCATACCACCGGATCT-3'; CD155 forward, 5'-TATTCGGAGTCCAAACGGCT-3'; CD155 reverse, 5'-CTTCATCCTCTACGCGCAAC-3'; PSGL1 forward, 5'-GGAACCCCTGAGTCTACCAC-3'; PSGL1 reverse, 5'-ATAGCTGCTGAATCCGTGGA-3'; CEACAM1 forward, 5'-AAGCCCCAAATCAAAGCCAG-3'; CEACAM1 reverse, 5'-GACAGCTTCATCCTCTCCGA-3'; IGF2BP2 forward, 5'-AGCTAAGCGGGCATCAGTTTG-3'; IGF2BP2 reverse, 5'-CCGCAGCGGGAAATCAATCT-3'; BRCC3 forward, 5'-GCACAGTTGCTGAAAAGGTTG-3'; BRCC3 reverse, 5'-TCGGTCCTTCCTCTTATCAGAAC-3'; GAPDH forward, 5'-ACCACCCTGTTGCTGTAGCCAA-3'; and GAPDH reverse, 5'-GTCTCCTCTGACTTCAACAGCG-3'.

### Immunofluorescence (IF) Staining

After seeding on glass slides, cells were fixed with 4% paraformaldehyde (PFA) for 15 min at room temperature, and then permeabilized with Triton X-100 in PBS for 10 min. For tissue samples, paraffin-embedded sections were deparaffinized in xylene, rehydrated through a graded ethanol series, and subjected to antigen retrieval in citrate buffer (pH 6.0) at 95 °C for 20 min. After blocking with 5% BSA for 1 hour, samples were incubated with primary antibodies overnight at 4 °C. On the following day, the samples were incubated for 1 h at room temperature with one of the following secondary antibodies (all from Abbkine, China; 1:200 dilution): Alexa Fluor 594-conjugated donkey anti-mouse IgG (A24411), IFKine Green Donkey Anti-Mouse IgG (A24211), IFKine Red Donkey Anti-Rabbit IgG (A24421), or IFKine Green Donkey Anti-Rabbit IgG (A24221). Nuclei were counterstained with 4′,6-diamidino-2-phenylindole (DAPI; BMU107-CN, Abbkine, China). Fluorescence images were captured on a Nikon T2 laser scanning confocal microscope equipped with appropriate filter sets. Primary antibodies used: Anti-IGF2BP2 (11601-1-AP, Proteintech, China, 1:100), Anti-LAMP2 (66301-1-Ig, Proteintech, China, 1:200) and Anti-CD8 (bs-0648R, Bioss, China, 1:100).

### Protein and RNA stability assays

To assess protein stability, cells were treated with cycloheximide (CHX; 100 μg/mL) and harvested at 0, 3, 6, and 9 h for Western blotting of IGF2BP2. To assess mRNA stability, cells were treated with actinomycin D (ActD; 5 μg/mL) and collected at 0, 4, 8, and 12 h for RT-qPCR analysis of PD-L1 mRNA. These assays were performed under the indicated SH2B3, BRCC3, IGF2BP2, or METTL3 manipulation conditions to evaluate rescue effects and alterations in transcript and protein half-lives.

### Co-immunoprecipitation (Co-IP) assay

NP-40 lysis buffer (Beyotime, China) containing protease inhibitors was used to lyse the cells. After centrifugation, the supernatants were collected and incubated with the indicated antibodies (anti-SH2B3, anti-IGF2BP2, anti-BRCC3, or control IgG; 4 μg/mL for IP antibodies) overnight at 4 °C, followed by incubation with protein A/G beads (HY-K0202, MCE, USA) for 3 h at 4 °C. When indicated, lysates were treated with RNase A to exclude RNA-dependent interactions. After washing with precooled PBS, the immunocomplexes were eluted by boiling in loading buffer and analyzed by Western blotting. For exogenous interactions, 293T cells were cotransfected with the indicated plasmids and harvested 48 h later before IP-WB analysis.

### Ubiquitination assay

For K63-linked ubiquitination detection, cells were treated with CQ (50 μM) for 24 h before harvest. For total ubiquitination and K48-linked ubiquitination detection, cells were treated with CQ (50 μM) for 24 h, with MG132 (10 μM) added during the last 6 h before harvest. For denaturing ubiquitination immunoprecipitation, cells were lysed in RIPA buffer containing 1% SDS, 10 mM N-ethylmaleimide (NEM), and protease inhibitors, followed by boiling at 95 °C for 10 min to disrupt non-covalent interactions. The lysates were then diluted tenfold with NP-40 buffer to reduce SDS to 0.1% before incubation with the indicated antibodies overnight at 4 °C. Protein A/G beads were added for an additional 3 h at 4 °C. After extensive washing, immunoprecipitates were analyzed by Western blotting using anti-ubiquitin, anti-K63-linked ubiquitin, or anti-K48-linked ubiquitin antibodies.

### Lyso-IP

Lysosomes were isolated using a TMEM192-tag-based lysosome immunoprecipitation (Lyso-IP) approach. Cells stably expressing 3×HA-TMEM192 were washed twice with ice-cold phosphate-buffered saline (PBS) and harvested in ice-cold potassium phosphate-buffered saline (KPBS; 136 mM KCl and 10 mM KH2PO4, pH 7.25) supplemented with protease and phosphatase inhibitor cocktails. The cell suspension was centrifuged at 1,000 × g for 2 min at 4 °C, and the pellet was resuspended in 800 μL of ice-cold KPBS. An aliquot of the suspension was retained as the whole-cell input control and mixed with 4 × SDS loading buffer. The remaining suspension was homogenized on ice using a pre-chilled 2-mL Dounce homogenizer with a tight pestle (15-20 strokes). The homogenate was centrifuged at 1,000 × g for 2 min at 4°C to remove nuclei and unbroken cells. The resulting post-nuclear supernatant was immediately transferred to a fresh pre-chilled tube for immunoprecipitation. For Lyso-IP, anti-HA antibody was pre-incubated with Protein A/G magnetic beads in ice-cold KPBS for 60 min at 4 °C with gentle rotation. The antibody-conjugated beads were washed with ice-cold KPBS and then added to the post-nuclear supernatant. The mixture was incubated at 4 °C with gentle rotation for 3 h to capture HA-tagged TMEM192-positive lysosomes. Subsequently, the beads were collected using a magnetic stand and washed three times with ice-cold KPBS. The immunoprecipitated lysosomal fractions were eluted by adding 1 × SDS loading buffer and heating at 95 °C for 5 min. The eluted proteins were then analyzed by western blotting.

### Mouse model

The Animal Ethics Committee of Yangzhou University approved all animal experiments (Approval No. 202411017). Female C57BL/6 mice, 6 weeks old, were housed under specific pathogen-free (SPF) conditions with controlled temperature and humidity and a regular light-dark cycle.

For the subcutaneous glioblastoma model, 3 × 10⁶ GL261 cells suspended in sterile PBS were subcutaneously injected into the right flank of each mouse. Tumor volume measurements were performed in a blinded manner. Tumor dimensions were measured every 5 days using vernier calipers, and tumor volume was calculated using the formula: 0.5 × length × width². For therapeutic intervention, etoposide phosphate (ETO) was administered intraperitoneally at a dose of 15 mg/kg every 3 days. Where indicated, anti-PD-L1 antibody was also administered intraperitoneally at 200 μg every 3 days.

For orthotopic glioma experiments, luciferase-expressing GL261 cells (1 × 10^5^ cells in 3 μL sterile PBS) were stereotactically implanted into the right striatum under isoflurane anesthesia at the following coordinates relative to bregma: AP, +0.5 mm; ML, +2.0 mm; and DV, -3.0 mm. Cells were injected at 0.4 μL/min, and the needle was retained in place for 5 min before slow withdrawal to minimize reflux. Tumor growth was monitored by bioluminescence imaging. On day 7 after tumor implantation, tumor establishment was confirmed by bioluminescence imaging, and mice were randomly allocated to treatment groups with comparable baseline bioluminescence signals. Mice received local convection-enhanced delivery (CED) of an isotype-matched control antibody or anti-mouse PD-L1 antibody at an equivalent dose (20 μg in 5 μL sterile PBS per injection). Etoposide phosphate (80 μM in 5 μL per mouse) or the corresponding vehicle was administered using the same schedule. CED was performed on day 7 and every 3 days thereafter for a total of four administrations.

For each CED procedure, mice were anesthetized with isoflurane, and infusions were delivered through the original burr hole at the tumor implantation site using identical stereotactic coordinates and a high-precision microinfusion pump. A total volume of 5 μL was infused at 0.4 μL/min. The needle was retained in place for 5 min after infusion and then slowly withdrawn to minimize reflux. The general health status, body weight, and neurological behaviors of mice were monitored daily throughout the experiment. Mice were humanely euthanized upon developing severe neurological dysfunction or losing more than 20% of initial body weight. At the experimental endpoint, mice were perfused with pre-chilled sterile PBS, and brain tissues and tumor samples were harvested for subsequent flow cytometry, immunohistochemistry, and immunofluorescence analyses.

### Flow cytometry

Following intracardiac perfusion with ice-cold phosphate-buffered saline (PBS), tumor tissues were excised, mechanically minced, and enzymatically digested in collagenase/DNase-containing digestion buffer to generate single-cell suspensions. The digested tissues were sequentially filtered through 100-μm and 70-μm cell strainers. Cells were then resuspended in complete RPMI-1640 medium. Before antibody staining, cells were incubated with BeyoFC™ Fc Receptor Blocking Solution (anti-mouse CD16/CD32; Cat# C1755M, Beyotime, China; 1:100) on ice for 10 min to reduce nonspecific Fc receptor-mediated antibody binding. Dead cells were excluded using Fixable Viability Dye eFluor™ 780 (Cat# 65-0865-18, eBioscience, USA; 1:1000) or Zombie Violet™ Fixable Viability Kit (Cat # 423113, BioLegend, USA; 1:1000). Surface staining was performed on ice for 30 min in the dark. For intracellular cytokine and granzyme B staining, cells were stimulated *ex vivo* with phorbol 12-myristate 13-acetate (PMA) and ionomycin in the presence of a protein transport inhibitor for 4 h at 37 °C. Following stimulation, cells were stained for surface markers, fixed and permeabilized according to the manufacturer's instructions, and subsequently stained with intracellular antibodies. PD-1 and TIM-3 were stained as surface markers before fixation and permeabilization. To minimize spectral overlap and facilitate reproducible analysis, cells from each sample were divided into separate aliquots and stained using dedicated antibody panels. Compensation was established using single-stained controls, and fluorescence-minus-one controls were included where appropriate. T-cell infiltration was assessed by identifying live CD45^+^CD3^+^CD8^+^ cells. This panel included Fixable Viability Dye eFluor™ 780, anti-CD45 Pacific Blue 450 (PB450) (Cat# 103126, BioLegend, USA; 1:200), anti-CD3 APC (Cat# 100242, BioLegend, USA; 1:200), and anti-CD8a PE (Cat# 100712, BioLegend, USA; 1:200). For assessment of CD8 T-cell effector function, live CD45^+^CD3^+^CD8^+^ T cells were analyzed for intracellular IFN-γ or granzyme B (GZMB) expression. The backbone markers included Fixable Viability Dye eFluor™ 780, anti-CD45 BV510 (Cat# 103137, BioLegend, USA; 1:200), anti-CD3 APC (Cat# 100242, BioLegend, USA; 1:200), and anti-CD8a Pacific Blue 450 (PB450) (Cat# 100725, BioLegend, USA; 1:200). Intracellular IFN-γ and GZMB were detected using anti-IFN-γ FITC (Cat# 505806, BioLegend, USA; 1:100) and anti-GZMB PE (Cat# 372208, BioLegend, USA; 1:100), respectively. For assessment of CD8 T-cell exhaustion, live CD45^+^CD3^+^CD8^+^ T cells were evaluated for surface PD-1 and TIM-3 expression using anti-PD-1 FITC (Cat# 135213, BioLegend, USA; 1:100) and anti-TIM-3 PE (Cat# 134004, BioLegend, USA; 1:100). Myeloid-derived suppressor cells (MDSCs) were identified as live CD45^+^CD11b^+^Gr-1^+^ cells. This panel included Fixable Viability Dye eFluor™ 780, anti-CD45 Pacific Blue 450 (PB450) (Cat# 103126, BioLegend, USA; 1:200), anti-CD11b FITC (Cat# 101206, BioLegend, USA; 1:100), and anti-Gr-1 (Ly-6G/Ly-6C) APC (Cat# 108412, BioLegend, USA; 1:200). macrophage populations were identified as live CD45^+^CD11b^+^F4/80^+^ cells. The macrophage panel included Zombie Violet™ Fixable Viability Kit (Cat# 423113, BioLegend, USA; 1:1000), anti-CD45 BV510 (Cat# 103137, BioLegend, USA; 1:200), anti-CD11b FITC (Cat# 101206, BioLegend, USA; 1:100), and anti-F4/80 PE (Cat# 123109, BioLegend, USA; 1:100). Macrophage-associated markers were assessed in separate staining tubes. CD86 was detected using anti-CD86 PE/Cyanine7 (Cat# 105013, BioLegend, USA; 1:200), whereas CD206 was detected using anti-CD206 APC (Cat# 141708, BioLegend, USA; 1:100). For intracellular cytokine and granzyme B staining, cells were fixed, permeabilized, and incubated with the corresponding antibodies according to the manufacturer's instructions. Data were acquired on a Beckman CytoFLEX flow cytometer and analyzed using FlowJo v10 software. Flow-cytometric acquisition and analysis were performed by investigators blinded to group allocation.

### Methylated RNA immunoprecipitation (MeRIP)-qPCR

For the MeRIP assay, total RNA was extracted from differentially treated GBM cells using TRIzol reagent. mRNA was then isolated with the Dynabeads mRNA Purification Kit (61006, Invitrogen, USA), fragmented, and immunoprecipitated with an anti-m⁶A antibody using the Magna MeRIP m⁶A Kit (A-P-9018, IVDSHOW, China). m⁶A-enriched mRNAs were subsequently analyzed by RT-qPCR using the following primers: PD-L1 Primer 1 Forward, GGTTGTGGATCCAGTCACCT, Reverse, GACTTCGGCCTTGGGGTAG. PD-L1 Primer 2 Forward, TGACAAGAGGAAGGAATGGGC, Reverse, CTGCTTTCGCCAGGTTCCAT.

### RNA immunoprecipitation (RIP)

RIP assays were performed using the EZ-Magna RIP Kit (Millipore, Burlington, MA, USA) according to the manufacturer's instructions. Cell lysates were incubated overnight at 4 °C on a rotator with magnetic beads coupled to anti-IGF2BP2 antibody or control IgG. After five washes with RIP wash buffer, the RNA-protein complexes were digested with Proteinase K, and the co-precipitated RNA was purified using spin columns. The purified RNA was reverse-transcribed and subjected to RT-qPCR analysis. The enrichment of target transcripts was calculated as percentage of input (% Input).

### IHC staining

Immunohistochemical staining was used to evaluate protein expression in tumor tissues. In brief, tumor tissue was fixed with 4% paraformaldehyde and paraffin-embedded. Following sectioning, the tumor tissue was deparaffinized, rehydrated, washed, and incubated overnight at 4 °C with specific primary antibodies. The following day, after washing, the sections were incubated with secondary antibodies. Immunoreactivity was visualized with 3,3'-diaminobenzidine (DAB). The nuclei were counterstained with hematoxylin. The samples were then examined under a microscope. Primary antibodies used: Anti-SH2B3 (DF9898, Affinity, China), Anti-PD-L1 (66248-1-Ig, Proteintech, China), Anti-IGF2BP2 (11601-1-AP, Proteintech, China) and Anti-Ki67 (ab16667, Abcam, USA).

### Microscale thermophoresis (MST)

The binding affinity between ETO and SH2B3 was quantified by MST using a Monolith NT.115 instrument (NanoTemper Technologies, Munich, Germany). Recombinant human SH2B3 protein was expressed and purified via affinity chromatography. Purified SH2B3 was fluorescently labeled with the Monolith Protein Labeling Kit RED-NHS 2nd Generation (NanoTemper Technologies, MO-L011) according to the manufacturer's instructions. The labeled protein was diluted to a final concentration of 50 nM in MST assay buffer (20 mM HEPES, 150 mM NaCl, 0.05% Tween-20, pH 7.4). ETO was serially diluted (2-fold) in the same buffer to generate a concentration gradient. Equal volumes of labeled SH2B3 and ETO dilutions were mixed and incubated at room temperature for 10 min in the dark. Samples were loaded into standard capillaries and MST measurements were performed at ambient temperature with 5% LED power and medium MST power. Binding curves and dissociation constants (Kd) were derived by fitting the normalized fluorescence data using MO.Affinity Analysis software (NanoTemper Technologies). Each experiment was performed in at least three independent replicates.

### GST pull-down assay

Recombinant GST-tagged SH2B3 and GST alone (as a negative control) were expressed in *Escherichia coli* BL21(DE3) and purified using GST resin (Beyotime, Shanghai, China). Recombinant His-tagged IGF2BP2 was expressed in *E. coli* BL21(DE3) and purified via Ni-NTA affinity chromatography. Purified GST or GST-SH2B3 proteins were immobilized on Glutathione Sepharose beads (Promega, Madison, WI, USA) and incubated with purified His-IGF2BP2 protein overnight at 4 °C. After extensive washing, bound proteins were eluted with SDS loading buffer and analyzed by immunoblotting using anti-GST and anti-His antibodies.

### Cellular Thermal Shift Assay (CETSA)

CETSA was performed to evaluate the thermal stabilization of endogenous SH2B3 upon ETO treatment. T98G cells were treated with ETO at the indicated concentrations or vehicle control overnight at 37 °C. Following treatment, cells were lysed in a non-denaturing buffer using repeated freeze-thaw cycles. The cleared lysates were divided into nine aliquots and subjected to a thermal gradient (37, 42, 47, 52, 57, 62, 67, 72, and 77 °C) for 3 min to induce protein denaturation, followed by high-speed centrifugation at 20,000 × g for 20 min at 4 °C to pellet the aggregated proteins. The supernatants containing soluble protein fractions were collected and analyzed by immunoblotting using an anti-SH2B3 antibody. The amount of soluble SH2B3 remaining at each temperature was quantified by densitometry and normalized to the signal at 37 °C (set as 100%). Thermal stability curves were generated by plotting the percentage of soluble protein versus temperature. Each experiment was performed in at least three independent replicates.

### Structure-based virtual screening targeting SH2B3

The crystal structure of SH2B3 was obtained from the Protein Data Bank (PDB entry 7R8W). Structure-based virtual screening was performed with MOE2019 software against the Drug Repurposing Compound Library Plus (MedChemExpress, HY-L035P). This library includes 6,320 approved drugs and compounds beyond Phase I clinical trials, all of which have undergone extensive preclinical and clinical studies and exhibit good bioactivity, safety, and bioavailability. The energy minimization routine in MOE2019 was applied to optimize the geometry of molecules within the library, and the refined library was then subjected to the screening protocol. To estimate binding affinity, the GBVI/WSA dG scoring function was adopted. Through this scoring-based ranking, ETO was ultimately singled out as a promising hit candidate. The structural visualizations were generated using UCSF ChimeraX.

### Plasmids, RNA interference, and transfection

Short hairpin RNA (shRNA), overexpression plasmids and wild-type plasmids were constructed by Nanjing Linghao Gene Biotechnology Co., Ltd. Briefly, polymerase chain reaction (PCR)-amplified human and mouse SH2B3, BRCC3, and IGF2BP2 were cloned and inserted into the pLV-CMV-MCS-puro-3×Flag vector, pLV-CMV-MCS-puro-Myc or pLV-CMV-MCS-puro-6×his vector. The shRNA target sequences were subsequently cloned and inserted into the PLKO.1-puro vector. HEK-293T cells were cotransfected with the recombinant plasmids and packaging plasmids using Polyplus according to the manufacturer's instructions (Sartorius, Germany). At 24 to 48 h later, the lentiviral particles were collected and stored at -80°C. For stable cell line construction, cells were infected with lentivirus in culture medium supplemented with 5 μg/mL polybrene (Beyotime Biotechnology, China) for 24 h. The cells were treated with puromycin (Biotechnology, China) for positive clone selection. The gene expression efficiency was determined by quantitative reverse transcription PCR (RT-qPCR) or immunoblot analyses.

The following shRNA sequences were used: sh-SH2B3 homo: sense, GGGCCATAGACAATCAGTACA and antisense, TGTACTGATTGTCTATGGCCC. sh-Sh2b3 mus: sense, CCTGACAACCTCTACACCTTT and antisense, AAAGGTGTAGAGGTTGTCAGG. sh-PD-L1 homo: sense, TTGACTCCATCTTTCTTCA and antisense, TGAAGAAAGATGGAGTCAA. sh-PD-L1 mus: sense, GGTGCGGACTACAAGCGAA and antisense, TTCGCTTGTAGTCCGCACC. sh-BRCC3 homo: sense, CCTGTTTCATAGAAGATAA and antisense, TTATCTTCTATGAAACAGG. sh-IGF2BP2 homo: sense, GCATGATTCTTGAAATCAT and antisense, ATGATTTCAAGAATCATGC. sh-Igf2bp2 mus: sense, GCCGCATGATTCTTGAGATTA and antisense, TAATCTCAAGAATCATGCGGC, sh-METTL3 homo: sense, GCAAGAATTCTGTGACTAT and antisense, ATAGTCACAGAATTCTTGC.

### Statistical analysis

Continuous variables were compared using an unpaired two-tailed Student's t test for two groups or one-way/two-way ANOVA with the appropriate post hoc test for multiple groups, as indicated in the figure legends. Data are presented as mean ± SD. Biological replicate numbers are specified in each figure legend, and technical replicates were used where appropriate. Survival analysis was performed using the Kaplan-Meier method. All statistical analyses were conducted using GraphPad Prism 8.0. *P* < 0.05 was defined as statistically significant.

## Results

### SH2B3 is upregulated in glioma and is associated with poorer survival

Database analyses revealed that SH2B3 mRNA expression was markedly elevated in glioma tissues compared with normal brain, and increased progressively with WHO grade **(Figure 1A, B)**. Survival analyses in both the TCGA and CGGA cohorts demonstrated that high SH2B3 mRNA expression was significantly associated with unfavorable clinical outcomes **(Figure 1C, D)**. Immunohistochemical (IHC) staining of clinical glioma samples further confirmed elevated SH2B3 protein levels, which positively correlated with WHO grade but showed no significant association with patient age or sex** (Figure 1E, Table 1)**. Consistently, Western blotting of paired glioma and peritumoral tissues validated a prominent increase of SH2B3 protein in tumor tissues **(Figure 1F)**. At the cellular level, both SH2B3 mRNA and protein were substantially upregulated in multiple human glioblastoma cell lines compared with human astrocytes (HA). Among glioblastoma cell lines, U251 showed the highest levels, whereas T98G showed relatively lower expression. **(Figure 1G, H)**.

### SH2B3 promotes glioblastoma progression *in vivo* but does not affect proliferation, migration, or invasion *in vitro*

To define the functional role of SH2B3 in glioblastoma, we generated GL261 cells with stable SH2B3 knockdown or overexpression and confirmed efficient modulation of SH2B3 expression **(Figure 2A, B)**. In C57BL/6 mice bearing subcutaneous tumors, SH2B3 depletion markedly reduced tumor size, volume, weight, and Ki67 positivity, whereas SH2B3 overexpression exerted the opposite effects **(Figure 2C, D)**. Consistently, in orthotopic glioblastoma models, SH2B3 knockdown significantly impaired intracranial tumor growth, while SH2B3 overexpression robustly accelerated tumor progression **(Figure 2E, F)**.

To dissect the underlying mechanisms, we knocked down SH2B3 in U251 cells (high endogenous SH2B3) and overexpressed SH2B3 in T98G cells (low endogenous SH2B3). After validating the stable cell lines, we performed EdU incorporation, CCK-8, wound-healing, and Transwell assays. Unexpectedly, SH2B3 modulation did not significantly influence cell proliferation, migration, or invasion *in vitro* **(Figure 2G-J, S1A-E)**. Similar results were obtained in GL261 cells **(Figure S1F-J)**. These findings suggest that the tumor-promoting effects of SH2B3 may be related to the tumor microenvironment, which is not fully recapitulated by conventional two-dimensional assays.

### SH2B3 upregulates PD-L1 expression in glioblastoma cells

To investigate how SH2B3 promotes glioblastoma progression, we performed transcriptome profiling following SH2B3 knockdown. RNA-seq analysis revealed PD-L1, a key immune checkpoint molecule involved in tumor immune evasion, as one of the most significantly downregulated genes **(Figure 3A-C)**. We further examined the expression of several immune-related molecules, including PD-L1, CD24, CD47, CD155, PSGL1, and CEACAM1, via qRT-PCR. The results demonstrated that PD-L1 expression was markedly reduced upon SH2B3 knockdown and significantly elevated upon SH2B3 overexpression (Figure 3D, E). For the other candidates, SH2B3 knockdown significantly downregulated CD47 and CEACAM1 while upregulating CD155 and PSGL1, but showed no significant effect on CD24. In contrast, SH2B3 overexpression led to a significant decrease in CD24 expression, whereas the levels of CD47, CD155, PSGL1, and CEACAM1 remained unchanged **(Figure 3D, E)**. Among these molecules, PD-L1 displayed the most consistent and robust bidirectional response to SH2B3 modulation; therefore, we prioritized PD-L1 for subsequent mechanistic analyses. We further confirmed by Western blotting and flow cytometry that SH2B3 promotes PD-L1 expression** (Figure 3F-G)**. Similar results were obtained in GL261 cells **(Figure S2A)**. In both the TCGA and CGGA datasets, SH2B3 expression positively correlated with PD-L1 levels **(Figure 3H)**. IHC staining of mouse tumor tissues further confirmed that SH2B3 upregulates PD-L1 *in vivo* **(Figure 3I)**. Collectively, these results indicate that SH2B3 promotes PD-L1 expression in glioblastoma cells.

### SH2B3 drives glioblastoma progression *in vivo* by enhancing PD-L1-mediated immune evasion

Given that PD-L1 suppresses T cell activity via PD-1 engagement, we quantified CD8⁺ T cell infiltration and IFN-γ⁺CD8⁺ T cell frequency in mouse tumors. SH2B3 knockdown increased, whereas SH2B3 overexpression reduced, both tumor-infiltrating CD8⁺ T cells and the proportion of IFN-γ-producing CD8⁺ T cells, indicating that SH2B3 impairs antitumor immunity **(Figure S3A-D)**.

To determine whether PD-L1 mediates SH2B3-driven tumor progression, we silenced PD-L1 in SH2B3-overexpressing GL261 cells** (Figure S3E)**. PD-L1 knockdown markedly reversed the enhanced tumor growth induced by SH2B3 overexpression *in vivo* **(Figure 4A)**. Furthermore, treatment with a PD-L1 monoclonal antibody markedly attenuated the pro-tumorigenic effects of SH2B3 overexpression and reduced Ki67 expression while restoring CD8⁺ T cell infiltration and IFN-γ⁺CD8⁺ T cell frequency to levels comparable to controls **(Figure 4B, S3F-I)**. These findings demonstrate that SH2B3 promotes glioblastoma progression largely by augmenting PD-L1-dependent immune evasion.

To better recapitulate the native central nervous system tumor microenvironment, we established orthotopic intracranial glioblastoma models **(Figure 4C)**. Bioluminescence imaging and Kaplan-Meier survival curves showed that SH2B3 overexpression aggravated intracranial tumor burden and shortened mouse survival, whereas anti-PD-L1 monoclonal antibody treatment largely reversed these malignant phenotypes **(Figure 4C-D)**. Flow cytometric analysis of orthotopic tumor tissues showed that SH2B3 overexpression increased the proportions of CD11b⁺Gr-1⁺ MDSCs and CD11b⁺F4/80⁺ macrophages while reducing the proportion of CD8⁺ T cells among CD45⁺ immune cells; these changes were largely reversed by anti-PD-L1 blockade** (Figure 4E)**. In parallel, SH2B3 overexpression decreased IFN-γ⁺ and GZMB⁺CD8⁺ T cells, increased TIM3⁺PD-1⁺ exhausted CD8⁺ T cells, and promoted an immunosuppressive macrophage phenotype characterized by elevated CD206 and reduced CD86 expression, all of which were significantly rescued by anti-PD-L1 monoclonal antibody treatment **(Figure S4A-H)**.

### SH2B3 enhances PD-L1 mRNA stability via IGF2BP2

To elucidate how SH2B3 elevates PD-L1 expression, we first examined PD-L1 mRNA stability. SH2B3 overexpression markedly prolonged the half-life of PD-L1 mRNA, suggesting post-transcriptional regulation **(Figure 5A)**. To identify proteins potentially involved in SH2B3-mediated post-transcriptional regulation, we performed SH2B3 immunoprecipitation followed by mass spectrometry. The leading SH2B3-associated proteins were ranked according to their Sum PEP Scores and are listed in Supplementary Table S1. These hits included proteins associated with RNA metabolism and modification, DNA damage repair, cytoskeletal organization, protein folding, and mitochondrial function. Among the proteins potentially involved in mRNA stability regulation, NSUN2 and IGF2BP2 were of particular interest. NSUN2 is a well-characterized m5C methyltransferase that has been implicated in RNA stability regulation, whereas IGF2BP2 is a canonical m⁶A reader known to stabilize target transcripts. Based on the identification of these RNA modification-related proteins, we performed *in silico* prediction of both m5C and m⁶A modification sites within PD-L1 mRNA. The predicted m5C sites showed relatively low confidence scores, whereas PD-L1 mRNA contained several higher-confidence predicted m⁶A sites. Meanwhile, MeRIP-qPCR revealed that PD-L1 transcripts pulled down by anti-m⁶A antibody were more abundant in SH2B3-overexpressing cells, supporting the involvement of an m⁶A-related post-transcriptional regulatory mechanism **(Figure 5B)**.

While several other high-ranking candidates were identified, IGF2BP2 was most directly linked to m⁶A-dependent mRNA stabilization and was therefore prioritized for functional validation. Therefore, we next examined whether IGF2BP2 was regulated by SH2B3. Indeed, SH2B3 overexpression selectively increased IGF2BP2 protein levels, whereas SH2B3 knockdown reduced them **(Figure 5C, D, S5A)**. Western blotting of clinical glioma specimens demonstrated a modest positive correlation between SH2B3 and IGF2BP2 protein levels** (Figure 5E)**. RIP assays using an IGF2BP2 antibody confirmed that IGF2BP2 binds PD-L1 mRNA, and this interaction was enhanced by SH2B3 overexpression but reduced by SH2B3 knockdown **(Figure 5F)**. To further test whether this regulation depends on m⁶A writing, we knocked down METTL3 in SH2B3-overexpressing cells** (Figure S5B)**. Under these conditions, the SH2B3-induced increase in PD-L1 mRNA stability was markedly attenuated. These data support the conclusion that SH2B3 enhances PD-L1 expression through an m⁶A-dependent transcript recognition mechanism rather than by directly altering m⁶A deposition.

Functionally, IGF2BP2 knockdown markedly attenuated SH2B3-induced PD-L1 upregulation, whereas IGF2BP2 overexpression rescued PD-L1 expression that was suppressed by SH2B3 knockdown **(Figure 5G, S5C)**. Together, these data indicate that SH2B3 enhances PD-L1 mRNA stability through IGF2BP2 in an m⁶A-dependent manner.

### SH2B3 prevents autophagy-lysosomal degradation of IGF2BP2

To investigate the molecular mechanism by which SH2B3 promotes IGF2BP2 upregulation, we first quantified IGF2BP2 mRNA levels and found that SH2B3 manipulation did not significantly alter its transcription** (Figure 6A)**. Given that SH2B3 affected IGF2BP2 protein abundance without influencing its mRNA expression, we next examined IGF2BP2 protein turnover. Cycloheximide chase assays showed that SH2B3 knockdown accelerated IGF2BP2 degradation, whereas SH2B3 overexpression markedly prolonged IGF2BP2 protein half-life **(Figure 6B)**.

To define the degradation pathway, we treated cells with MG132 (proteasome inhibitor), chloroquine (CQ), or 3-methyladenine (3-MA) (autophagy-lysosome inhibitors) 40. CQ and 3-MA largely abolished the SH2B3-dependent differences in IGF2BP2 protein abundance, whereas MG132 caused only a modest increase and failed to eliminate these differences. These findings indicate that SH2B3 primarily protects IGF2BP2 from autophagy-lysosome degradation rather than proteasomal degradation** (Figure 6C)**.

Lyso-IP assays in U251 cells revealed that SH2B3 knockdown markedly increased lysosomal accumulation of IGF2BP2** (Figure 6D)**. Consistently, immunofluorescence staining in U251 cells showed enhanced colocalization of IGF2BP2 with the lysosomal marker LAMP2 after SH2B3 knockdown, particularly under EBSS (an autophagy agonist‌)/CQ treatment **(Figure 6E)**. Together, these data demonstrate that SH2B3 inhibits lysosomal degradation of IGF2BP2.

### SH2B3 suppresses K63-linked polyubiquitination of IGF2BP2

K63-linked polyubiquitination is commonly involved in cargo sorting and autophagy-lysosome degradation of client proteins 29. We therefore examined IGF2BP2 ubiquitination patterns. SH2B3 knockdown significantly increased total and K63-linked ubiquitination of IGF2BP2, whereas K48-linked ubiquitination remained unchanged. Conversely, SH2B3 overexpression reduced overall ubiquitination and specifically decreased K63-linked polyubiquitination of IGF2BP2 **(Figure 7A, B)**.

To validate these findings, we conducted ubiquitination assays using K63-Ub and K63R-Ub mutants in HEK293T cells. SH2B3 overexpression failed to reduce IGF2BP2 ubiquitination in the presence of the K63R-Ub mutant, indicating that SH2B3 acts specifically on K63-linked ubiquitin chains** (Figure 7C, D)**. To determine whether IGF2BP2 undergoes ubiquitin-mediated degradation via the autophagy-lysosome pathway, we further used Bafilomycin A1 (Baf) to block autophagosome-lysosome fusion and EBSS to induce autophagy. Both treatments increased IGF2BP2 ubiquitination **(Figure 7E)**. Molecular docking analysis predicted a potential interaction interface between IGF2BP2 and the SH2 domain of SH2B3 **(Figure 7F)**. We constructed SH2B3 mutants lacking either the PH or SH2 domain **(Figure 7G)**. Co-immunoprecipitation assays showed that the SH2 domain, but not the PH domain, was required for SH2B3-IGF2BP2 interaction **(Figure 7H)**. Moreover, SH2B3 mutants lacking the SH2 domain (ΔSH2) lost the ability to suppress IGF2BP2 K63-linked ubiquitination **(Figure 7I-K)**. Consistently, GST pull-down assays with purified recombinant proteins further verified the direct *in vitro* binding between SH2B3 and IGF2BP2** (Figure 7L).** Collectively, GST pull-down assays support a direct interaction between SH2B3 and IGF2BP2 *in vitro*, whereas cellular truncation assays indicate that the SH2 domain is required for efficient SH2B3-IGF2BP2 association in cells.

### SH2B3 promotes BRCC3-mediated removal of K63-linked ubiquitin from IGF2BP2

The ubiquitination status of a protein is largely governed by E3 ubiquitin ligases and deubiquitinases (DUBs) 30. On this basis, we performed IP-MS to identify ubiquitin-modifying enzymes that associate with IGF2BP2 and SH2B3. Among the candidates, BRCC3 was identified as a biologically relevant interactor for further investigation **(Figure 8A)**. Previous work has shown that SH2B3 can regulate the BRCC3 isopeptidase complex (BRISC) to remove K63-linked polyubiquitin chains from JAK2, thereby limiting activation of the JAK2/STAT5 pathway 41. This led us to examine whether SH2B3 might similarly repress K63-linked polyubiquitination of IGF2BP2 through BRCC3. We first assessed BRCC3 mRNA and protein levels following SH2B3 knockdown or overexpression and found that SH2B3 did not alter BRCC3 expression **(Figure 8B, C)**. In contrast, BRCC3 silencing abrogated the SH2B3-induced upregulation of IGF2BP2 and PD-L1, while BRCC3 overexpression rescued IGF2BP2 and PD-L1 protein levels that were downregulated upon SH2B3 knockdown **(Figure 8D)**.

Notably, treatment with RNase A did not abolish the BRCC3-IGF2BP2 interaction, indicating that their association is largely independent of RNA. **(Figure S6A)**. Consistently, CHX chase assays showed that BRCC3 overexpression partly restored IGF2BP2 stability in SH2B3-knockdown U251 cells, whereas BRCC3 knockdown reduced IGF2BP2 stability in SH2B3-overexpressing T98G cells **(Figure S6B, C)**. Further ubiquitination assays demonstrated that SH2B3 attenuates K63-linked polyubiquitination of IGF2BP2 in a BRCC3-dependent manner** (Figure 8E)**. ActD chase experiments further showed that BRCC3 manipulation partially rescued the changes in PD-L1 mRNA stability caused by SH2B3 knockdown or overexpression **(Figure S6D, E)**, respectively, further supporting the SH2B3/BRCC3/IGF2BP2/PD-L1 regulatory cascade at the RNA stability level. Co-immunoprecipitation experiments further revealed that SH2B3 knockdown markedly weakened the interaction between BRCC3 and IGF2BP2, whereas SH2B3 overexpression enhanced their association** (Figure 8F)**. Given that SH2B3 has been reported to associate with BRCC3 through the BRISC adaptor KIAA0157 rather than by direct binding, the increased BRCC3-IGF2BP2 association observed following SH2B3 overexpression may be related to altered BRISC complex assembly or localization within IGF2BP2-containing protein complexes. Collectively, our findings are consistent with the possibility that SH2B3 modulates the BRCC3-IGF2BP2 association and may thereby contribute to BRCC3-associated reduction of K63-linked ubiquitination of IGF2BP2, IGF2BP2 stabilization, and PD-L1 upregulation. Further studies are needed to define the precise molecular basis of this regulation.

### ETO interferes with the SH2B3-mediated upregulation of IGF2BP2 and PD-L1

To explore a pharmacological strategy for targeting SH2B3, we screened small molecules predicted to bind SH2B3 **(Figure 9A)**. Screening results revealed that Etoposide phosphate (ETO) closely occupies the binding pocket of SH2B3, engaging in a dense network of polar interactions. Particularly, the negatively charged phosphate moiety of ETO forms robust salt bridges with the positively charged guanidinium groups of R343 and R364, which is presumed to be the predominant determinant of binding, and its interaction with SH2B3 was further supported by microscale thermophoresis (MST) and CETSA assays **(Figure 9B, C)**.

As a well-established topoisomerase II inhibitor, ETO is known to induce widespread DNA damage and non-specific cytotoxicity 42. This raised an important question: whether ETO-mediated repression of the SH2B3 axis results from specific target engagement or merely reflects a secondary consequence of general cellular toxicity. First, we generated SH2B3 constructs carrying single SH2-domain mutations, R343A or R364A, as well as the double mutation R343A/R364A **(Figure 9D, E)**. T98G cells expressing wild-type SH2B3, the single mutants R343A or R364A, or the double mutant R343A/R364A were treated with either vehicle or ETO. In the vehicle-treated groups, all SH2B3 variants significantly increased IGF2BP2 and PD-L1 expression to comparable levels, with no significant differences compared with wild-type SH2B3 overexpression, indicating that these mutations did not impair the intrinsic regulatory function of SH2B3. ETO markedly reduced IGF2BP2 and PD-L1 levels in cells expressing wild-type SH2B3, whereas these effects were substantially weakened in cells expressing the SH2B3 mutant **(Figure 9D, E)**. These results suggest that ETO may exert its effects through the R343/364 residues. In contrast, ETO caused comparable growth inhibition regardless of SH2B3 mutational status** (Figure 9F)**, consistent with its canonical topoisomerase II-dependent cytotoxic activity, which occurs independently of SH2B3. Together with our earlier observation that SH2B3 has minimal effects on glioblastoma cell proliferation under standard two-dimensional culture conditions, these findings indicate that ETO-mediated downregulation of IGF2BP2 and PD-L1 depends on intact R343/R364 residues, whereas its broad anti-proliferative effect reflects the intrinsic DNA-damaging activity of the drug.

Next, we treated glioblastoma cells with serially diluted doses of ETO **(Figure 9G-I)**. Immunoblot profiling revealed pronounced reductions in IGF2BP2 and PD-L1 at low concentrations, whereas detectable γ-H2AX induction was detected only at higher doses. CCK-8 assay revealed that ETO had limited impact on cell viability at low concentrations, whereas a marked reduction in cell viability was observed at higher doses. Together with the mutant-based validation described above, these data indicate that, at lower concentrations, ETO was associated with reduced IGF2BP2 and PD-L1 abundance before overt γ-H2AX induction under the tested conditions, whereas marked DNA damage signaling becomes prominent at higher doses. Ubiquitination assays further clarified the downstream molecular mechanism** (Figure 9J)**. ETO restored polyubiquitination of IGF2BP2, which was strongly suppressed by SH2B3 overexpression. These results explain how ETO disrupts SH2B3-mediated IGF2BP2 stabilization and the subsequent upregulation of PD-L1.

Collectively, these results are consistent with the possibility that ETO interferes with the SH2B3/IGF2BP2/PD-L1 axis in an SH2-domain-dependent manner. Given its concentration-dependent, SH2B3-independent cytotoxic effects, we define ETO in this manuscript as a preliminary exploratory lead compound rather than a highly selective SH2B3 inhibitor.

### ETO is associated with reduced tumor burden and altered immune profiles in orthotopic GL261 tumors

We first used subcutaneous tumor models as an *in vivo* system to evaluate the growth-promoting effect of SH2B3 and the therapeutic activity of ETO. SH2B3 overexpression (SH2B3-OE) enhanced subcutaneous tumor growth, whereas ETO treatment significantly reduced tumor volume and weight and largely abolished the SH2B3-driven growth advantage **(Supplementary Figure 7A-C)**.

We next established GL261 intracranial orthotopic glioblastoma models to further evaluate the antitumor and immunomodulatory effects of ETO in a brain tumor microenvironment **(Figure 10A)**. *In vivo* bioluminescence imaging revealed that SH2B3-OE substantially accelerated intracranial tumor expansion, whereas ETO markedly restrained tumor growth in both empty vector and SH2B3-OE tumors** (Figure 10A)**. Kaplan-Meier survival analysis further showed that SH2B3-OE shortened the survival of tumor-bearing mice, whereas ETO treatment prolonged survival and partially counteracted the survival disadvantage associated with SH2B3 overexpression** (Figure 10B)**.

Flow cytometric profiling of orthotopic tumors revealed that SH2B3-OE broadly reshaped the glioblastoma immune microenvironment toward an immunosuppressive state, characterized by reduced intratumoral CD8⁺ T-cell infiltration, decreased IFN-γ⁺ and GZMB⁺ effector CTL subsets, and increased PD-1⁺TIM-3⁺ exhausted CD8⁺ T cells. In parallel, SH2B3-OE expanded immunosuppressive CD11b⁺Gr-1⁺ myeloid-derived suppressor cells (MDSCs) and pro-tumoral CD11b⁺F4/80⁺ macrophage populations, with a shift toward a CD206^high^ M2-like macrophage phenotype. ETO treatment reversed these immune alterations, restoring CD8⁺ T cell infiltration and effector activity, reducing exhausted CD8⁺ T cells, limiting MDSC and macrophage populations accumulation, and redirecting macrophage populations toward a CD86^high^ state **(Figure 10C, S7D-K)**.

At the molecular level, immunoblotting of orthotopic tumor lysates showed that ETO reduced IGF2BP2 and PD-L1 abundance in orthotopic tumor tissues *in vivo*, while SH2B3 protein levels were not significantly changed **(Figure 10D)**, consistent with its ability to disrupt the SH2B3/IGF2BP2/PD-L1 axis in the orthotopic glioblastoma setting.

Finally, we performed IHC staining in human glioma specimens to assess the clinical relevance of the SH2B3/IGF2BP2/PD-L1 axis **(Figure 10E)**. Representative images showed stronger IGF2BP2 and PD-L1 staining in SH2B3-high tumors than in SH2B3-low tumors. Quantitative correlation analysis revealed positive associations between SH2B3 and IGF2BP2 scores (*r* = 0.6532, *P* = 0.0013), as well as between SH2B3 and PD-L1 scores (*r* = 0.8022, *P* < 0.0001), supporting the translational relevance of this regulatory cascade in human glioblastoma **(Figure 10E).**

## Discussion

SH2B3 was originally characterized as a multifunctional adaptor involved in cytokine and growth factor signaling; however, accumulating evidence now implicates it in diverse oncogenic processes across multiple tumor types 38. SH2B3 has been implicated in regulating key cancer-associated signaling pathways and may represent a potential therapeutic target 36, 37. In the present study, we demonstrate that SH2B3 is markedly upregulated in glioblastoma and functions as a tumor promoter primarily by enhancing PD-L1-mediated immune evasion. Mechanistically, our data are consistent with a model in which SH2B3 modulates the BRCC3-dependent K63-linked deubiquitination of IGF2BP2, thereby contributing to IGF2BP2 stabilization and increased PD-L1 expression **(Figure 11)**. Through this process, SH2B3 reinforces immune evasion and promotes glioblastoma progression. Investigating SH2B3's mechanism in glioblastoma and elucidating its specific pathway in regulating immune evasion provide novel therapeutic targets and conceptual frameworks for glioblastoma immunotherapy.

Immune evasion is widely recognized as a hallmark of malignant tumors 43, and the restoration of antitumor immunity by disrupting immune evasion mechanisms has reshaped cancer therapy 44. Immune checkpoint inhibitors targeting the PD-1/PD-L1 axis have demonstrated substantial survival benefits in melanoma, non-small cell lung cancer, renal cell carcinoma, and bladder cancer, where they are now incorporated into standard-of-care treatment regimens 45. These clinical successes highlight the therapeutic efficacy of inhibiting immune evasion, particularly the PD-1/PD-L1 axis. However, their limited efficacy in glioma underscores unique challenges, including poor BBB penetration, the profoundly immunosuppressive TME, and the pronounced heterogeneity of glioma cells 46. Against this backdrop, our elucidation of the SH2B3-driven PD-L1 regulatory pathway in glioblastoma provides not only key mechanistic insight into glioblastoma immune dysregulation but also identifies a therapeutically actionable node. Targeting SH2B3 or its downstream effectors may therefore capitalize on established successes in other cancer immunotherapies and help overcome the formidable immune barriers characteristic of glioma. Although our rescue experiments strongly support PD-L1 as a major downstream effector of SH2B3-mediated immune evasion, PD-L1 is unlikely to be the only relevant output of this pathway. The SH2B3/IGF2BP2 axis may also influence additional immune checkpoints or tumor-intrinsic programs that contribute to the overall immunosuppressive phenotype, which warrants further investigation.

PD-L1 is regulated through multiple nonredundant mechanisms at the transcriptional, post-transcriptional, and post-translational levels. For example, CMTM6 stabilizes PD-L1 protein at the cell surface, whereas COP1 has been implicated in transcriptional regulation of PD-L1 through the COP1/c-Jun/HDAC3 axis 47, 48. Our findings add a conceptually different layer to this regulatory network by linking post-translational control of an m⁶A reader protein to the stability of PD-L1 mRNA. In the highly immunosuppressive GBM microenvironment, this coupling may provide an efficient means of sustaining PD-L1 expression, because it simultaneously preserves the abundance of IGF2BP2 and enhances recognition of m⁶A-marked PD-L1 transcripts.

An important finding of this study is the identification of a novel pathway in which abnormally elevated SH2B3 level during glioblastoma progression upregulates PD-L1 expression through an IGF2BP2-mediated m⁶A-dependent transcript recognition and stabilization mechanism, offering a new perspective on immune evasion in glioblastoma. Consequently, inhibiting PD-L1 is crucial for exploring targeted therapies in glioblastoma. Currently, inflammatory cytokine stimulation, enhanced oncogenic signaling, post-translational modification regulation, and epigenetic regulation have all been demonstrated to regulate PD-L1 expression and membrane localization stability 49. This study is the first to demonstrate in glioblastoma that SH2B3 promotes PD-L1 mRNA stability and expression through an epigenetic regulatory pathway. mRNA stability is essential for maintaining translation efficiency and function, with mRNA modification pathways primarily including N6-methyladenosine (m⁶A), 5-methylcytidine (m5C), and 7-methylguanosine (m7G) 50. As a key molecule in immune evasion, PD-L1 can undergo m⁶A methylation modification, for example, circMYO1C targets an m⁶A site within PD-L1 mRNA and cooperates with IGF2BP2 to enhance its stability, thereby accelerating immune evasion in pancreatic ductal adenocarcinoma (PDAC) 51. IGF2BP2 binds to specific mRNA sequences to prevent mRNA degradation, prolonging its half-life and enhancing translation efficiency through interactions with the translational machinery, therefore, targeting IGF2BP2 to regulate PD-L1 represents a novel functional epigenetic pathway. In this study, IGF2BP2 overexpression significantly reversed SH2B3 knockdown-induced PD-L1 expression in glioblastoma cells, and both molecules showed high correlation in patient tissues. Given the interconnected nature of m⁶A-mediated gene regulation, this study identifies an mRNA regulatory mechanism involving IGF2BP2 and PD-L1, and demonstrates that targeting SH2B3, along with other molecular interventions, can produce anti-glioblastoma effects.

Previous studies have established that SH2B3 interacts with the BRISC complex rather than directly binding to BRCC3 itself 41. The BRISC (BRCC36 isopeptidase complex) is a cytoplasmic deubiquitinating enzyme complex that specifically cleaves K63-linked polyubiquitin chains; its core components include KIAA0157, BRCC36 (BRCC3), MERIT40, and BRCC45, with BRCC36 serving as the catalytic subunit. SH2B3 may recruit the entire BRISC complex to its substrates through its interaction with the unique BRISC component KIAA0157, thereby facilitating the removal of K63-linked ubiquitin chains from the substrate. Based on this mechanism, SH2B3 may also facilitate BRCC3-dependent regulation of IGF2BP2 ubiquitination by recruiting the BRISC complex and its catalytic subunit BRCC3 to the substrate IGF2BP2, rather than through direct binding between SH2B3 and BRCC3. However, this specific molecular scaffold model still requires further biochemical validation. Nevertheless, this inference is indirectly supported by our experimental data: SH2B3 knockdown did not alter the expression levels of BRCC3 itself, suggesting that SH2B3 functions by promoting the interaction between BRCC3 and IGF2BP2, rather than by modulating BRCC3 abundance. Additionally, we confirmed that purified recombinant SH2B3 and IGF2BP2 showed direct binding in a GST pull-down assay.

Notably, this study reveals that SH2B3 mediates BRCC3-dependent K63-linked ubiquitin chain removal of IGF2BP2. Consequently, BRCC3 inhibition emerges as an important avenue for the development of targeted glioblastoma therapies. BRCC3 has been implicated in distinct resistance-related contexts. In HNSCC, miR-21-mediated BRCC3 suppression inhibits NLRP3 inflammasome activation and is associated with cisplatin resistance 52. In prostate cancer models with acquired olaparib resistance, BRCC3 upregulation has been observed; however, its direct functional role in PARP inhibitor resistance remains to be clarified 53. Protein degradation primarily occurs through the ubiquitin-proteasome system and the autophagy-lysosomal pathway 26. As a key deubiquitinase, BRCC3 deubiquitinates ALK2 at Lys-472 and Lys-475, leading to downstream transcriptional activation 30. However, BRCC3-dependent regulation of IGF2BP2 ubiquitination by removing K63-linked ubiquitin chains through the autophagy-lysosomal pathway has never been reported. This work not only uncovers a previously unrecognized degradation mechanism for IGF2BP2 but also establishes SH2B3 as a key modulator of epigenetic pathways through BRCC3, thereby laying a foundation for future translational strategies targeting this axis in glioblastoma.

This study has several limitations. First, although we extended the *in vivo* validation using orthotopic glioblastoma models, further pharmacological validation with more selective SH2B3-targeting compounds is still needed. Second, the clinical significance of SH2B3 should be interpreted cautiously. Our cohort was relatively small and derived from a single center, and multivariable analyses incorporating key molecular features, including IDH status, MGMT methylation, and chromosome 7/10 alterations, were not performed. Accordingly, SH2B3 is more appropriately described as being associated with higher glioma grade and poorer survival rather than as a definitively established independent prognostic factor. Third, whether SH2B3 regulates additional post-translational modifications of IGF2BP2 remains unclear. Fourth, the relevance of this pathway to temozolomide resistance and other clinically important therapeutic contexts requires further investigation. Fifth, a notable discrepancy in our study is the apparent absence of SH2B3 effects on glioblastoma cell proliferation, migration, or invasion under standard two-dimensional culture conditions, despite its robust tumor-promoting activity *in vivo*. Our inference that SH2B3 primarily operates through immune- or microenvironment-dependent mechanisms, while reasonable, remains speculative. Several alternative explanations warrant consideration. First, the complex three-dimensional architecture, extracellular matrix interactions, and stromal cell contributions present *in vivo* are not recapitulated in conventional 2D assays, and SH2B3 may influence tumor growth indirectly by modulating the tumor microenvironment rather than by directly altering cell-intrinsic properties. Second, SH2B3 could regulate nutrient or metabolic stress responses that are only engaged under the physiological constraints of the *in vivo* setting. Third, compensatory signaling pathways activated *in vitro* may mask SH2B3-dependent phenotypes that become unmasked in the more physiologically demanding *in vivo* environment. Fourth, although our flow cytometric and immunohistochemical data point toward PD-L1-mediated immune evasion as a major downstream mechanism, we cannot exclude the possibility that additional SH2B3-dependent pathways, such as chemokine secretion, extracellular vesicle composition, or stromal cell reprogramming, also contribute to its *in vivo* protumorigenic effects. Future studies employing three-dimensional co-culture models, syngeneic immune-competent versus immunodeficient systems, or conditional knockout approaches will be necessary to delineate the relative contributions of tumor-autonomous versus microenvironment-dependent mechanisms to SH2B3-driven glioblastoma progression. Finally, our pharmacological data with ETO should be interpreted cautiously. ETO is a well-established Topoisomerase II inhibitor whose canonical antitumor effects are mainly mediated by DNA damage and cytotoxicity. Although our docking, MST, CETSA, ubiquitination, and SH2-domain mutant experiments support a SH2B3-related component of action, these findings do not justify classifying ETO as a selective SH2B3 inhibitor. Rather, ETO should be regarded here as a pharmacologically informative and translationally exploratory probe. The development of more selective SH2B3-targeting compounds will be essential for rigorously isolating and validating this mechanism in future studies.

In summary, this study elucidates a mechanism through which SH2B3 promotes IGF2BP2 deubiquitination and stabilization in a BRCC3-dependent manner, thereby enhancing m⁶A-dependent recognition and stabilization of PD-L1 transcripts and promoting immune evasion in glioblastoma. These findings provide important insight into the immunoevasive landscape of glioblastoma. While SH2B3 has been primarily studied for its role in regulating cellular signaling processes, our work demonstrates for the first time that glioblastoma-derived SH2B3 drives tumor progression through PD-L1-dependent immune evasion. Given that post-translational and epigenetic modifications are reversible and that inhibitors targeting these pathways are rapidly advancing toward clinical application, our findings support the translational potential of therapeutically targeting the SH2B3/BRCC3/IGF2BP2 axis in glioblastoma.

## Supplementary Material

Supplementary figures.

Supplementary table.

## Figures and Tables

**Figure 1:**
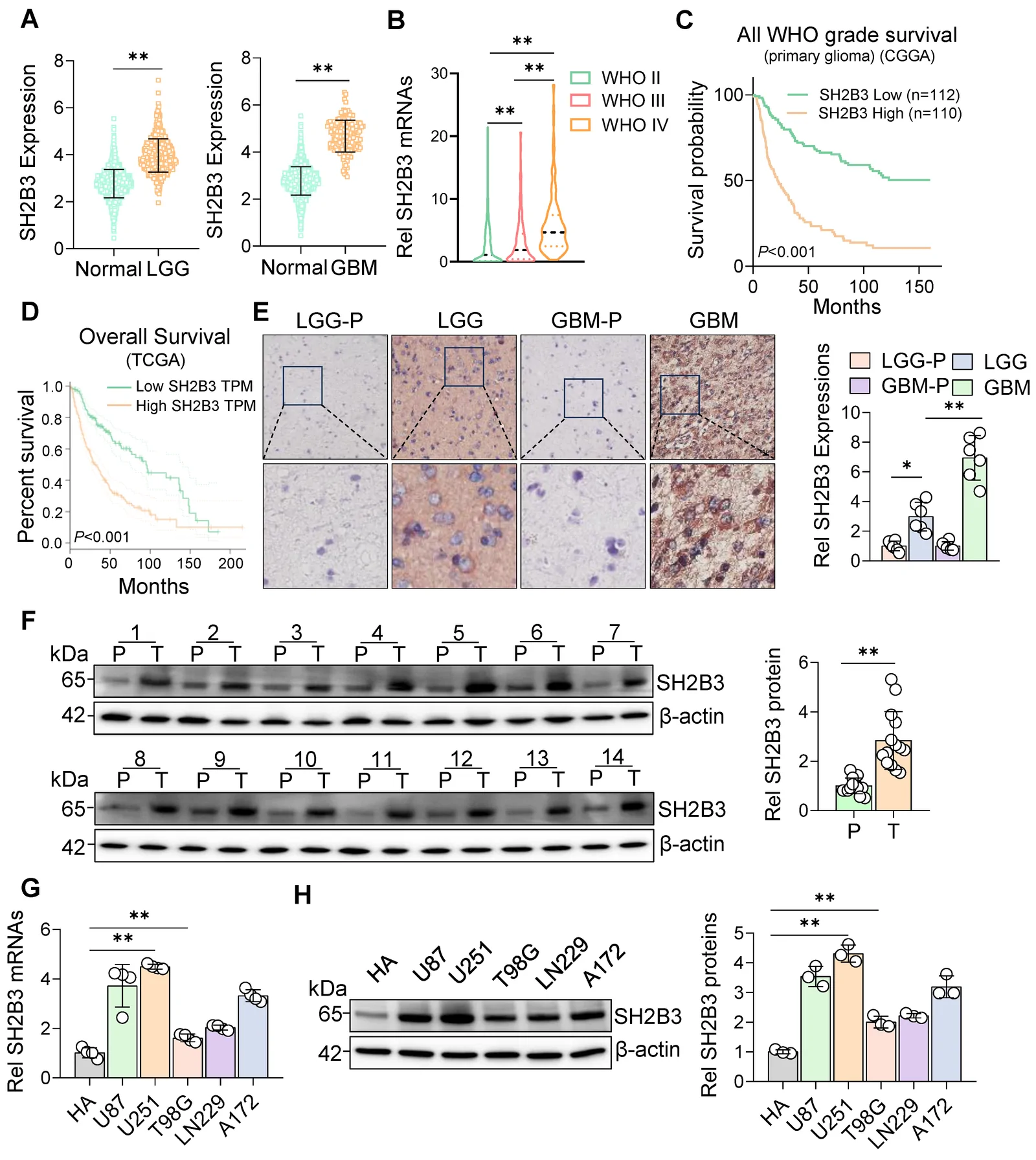
** SH2B3 is upregulated in glioma and is associated with poorer survival. A** SH2B3 expression in TCGA low-grade glioma (LGG) and glioblastoma (GBM) samples and GTEx normal brain samples, **P < 0.01. **B** The expression of SH2B3 in grade II, grade III, and grade IV glioma tissues was analyzed using CGGA database (**P < 0.01). **C** Kaplan-Meier survival analysis was performed on glioma patients using the CGGA database, with stratification by SH2B3 mRNA expression (***P < 0.001). **D** Kaplan-Meier survival curves for glioma patients were generated from the TCGA database, with stratification by SH2B3 expression (***P < 0.001). **E** Immunohistochemical staining of SH2B3 in LGG, GBM and peritumoral tissues and quantitative analysis (mean ± SD, *P < 0.05, **P < 0.01). **F** Western blotting of SH2B3 protein levels in paired tumor (T) and peritumoral tissue (P), with quantitative analysis (mean ± SD, **P < 0.01). **G** RT-qPCR was used to evaluate SH2B3 mRNA levels in HA, U87, U251, T98G, LN229, and A172 cells, and differential expression was assessed (n = 4, mean ± SD, **P < 0.01). **H** SH2B3 protein expression in HA, U87, U251, T98G, LN229, and A172 cells was detected by Western blotting and subsequently quantified (n = 3, mean ± SD, **P < 0.01).

**Figure 2:**
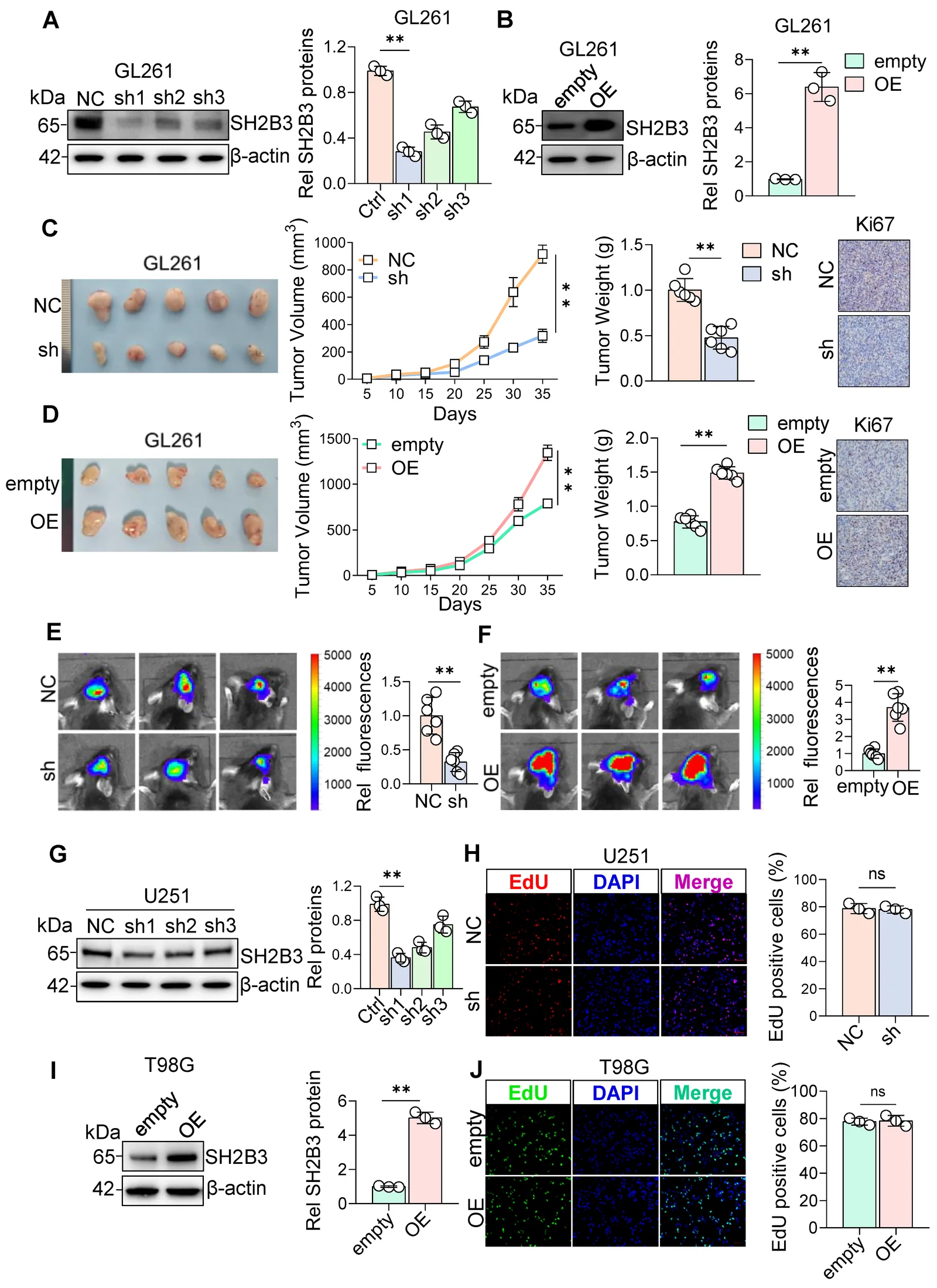
** SH2B3 promotes glioblastoma progression *in vivo* but does not affect proliferation *in vitro*. A** After transduction of GL261 cells with sh-SH2B3 lentivirus, the expression of SH2B3 protein was detected by Western blotting, and quantitative analysis was performed (n = 3, mean ± SD, **P < 0.01). **B** After transduction of GL261 cells with SH2B3-overexpressing lentivirus, the expression of SH2B3 protein was detected by Western blotting, and quantitative analysis was performed (n = 3, mean ± SD, **P < 0.01). **C** NC and sh-SH2B3 cells were injected subcutaneously into the flank of C57BL/6 mice to evaluate tumor growth *in vivo*. Representative excised tumor images. Tumor volume and tumor weight were assessed (n = 6, mean ± SD, **P < 0.01). Ki67 in tumor tissues was detected by Immunohistochemical staining. **D** Empty and OE-SH2B3 cells were injected subcutaneously into the flank of C57BL/6 mice to evaluate tumor growth *in vivo*. Representative excised tumor images. Tumor volume and tumor weight were assessed (n = 6, mean ± SD, **P < 0.01). Ki67 in tumor tissues was detected by Immunohistochemical staining. **E** Representative bioluminescence images showing luciferase signals in tumors in C57BL/6 mice in NC and sh-SH2B3 groups acquired by IVIS imaging, and quantitative analysis was performed (n = 6, mean ± SD, **P < 0.01). **F** Representative bioluminescence images showing luciferase signals in tumors in C57BL/6 mice in empty and OE-SH2B3 groups acquired by IVIS imaging, and quantitative analysis was performed (n = 6, mean ± SD, **P < 0.01). **G** Following shRNA transfection in U251 cells, SH2B3 protein expression was detected by Western blotting and quantified (n = 3, mean ± SD, **P < 0.01). **H** EdU staining assay was used to evaluate the changes of proliferation ability of U251 cells after SH2B3 knockdown (n = 3, mean ± SD, ns, nonsignificant). Scale bar, 100 μm. **I** After transfection with overexpressed lentivirus of T98G cells, the expression of SH2B3 protein was detected by Western blotting, and quantitative analysis was performed (n = 3, mean ± SD, **P < 0.01). **J** EdU staining assay was used to evaluate the changes of proliferation ability of T98G cells after SH2B3 overexpression (n = 3, mean ± SD, ns, nonsignificant). Scale bar, 100 μm.

**Figure 3:**
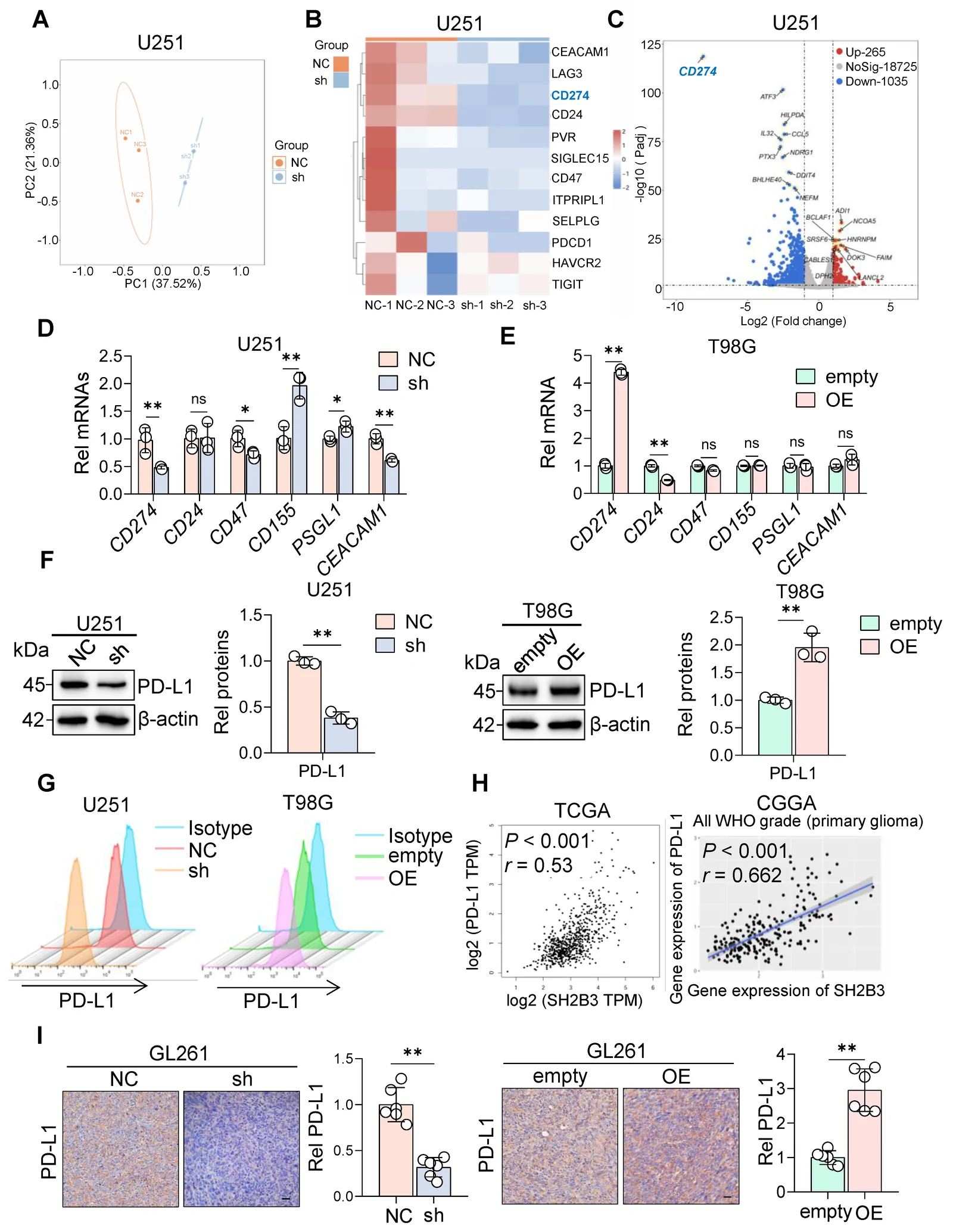
** SH2B3 upregulates PD-L1 expression in glioblastoma cells. A** Principal component analysis (PCA) of NC and sh-SH2B3 groups in U251 cells based on RNA-seq data. **B** Heatmap of differentially expressed genes between NC and sh-SH2B3 groups in U251 cells based on RNA-seq data. (n = 3 per group) **C** Volcano plot of differentially expressed genes between NC and sh-SH2B3 groups in U251 cells based on RNA-seq data. **D** The differential expression of *CD274, CD24, CD47, CD155, PSGL1* and *CEACAM1* mRNA in U251 cells after SH2B3 knockdown was analyzed by RT-qPCR (n = 3, mean ± SD, *P < 0.05, **P < 0.01, ns, nonsignificant). **E** The differential expression of *CD274, CD24, CD47, CD155, PSGL1* and *CEACAM1* mRNA in T98G cells after SH2B3 overexpression was analyzed by RT-qPCR (n = 3, mean ± SD, *P < 0.05, **P < 0.01, ns, nonsignificant). **F** Western blotting was used to detect PD-L1 protein expression in U251 cells after SH2B3 knockdown and in T98G cells after SH2B3 overexpression (n = 3, mean ± SD, **P < 0.01). **G** Flow cytometry detected the expression of PD-L1 in U251 cells after SH2B3 knockdown and in T98G cells after SH2B3 overexpression. **H** The correlation between SH2B3 and PD-L1 in glioma tissues was analyzed using the TCGA and CGGA databases. **I** Immunohistochemical staining of PD-L1 in GL261 mouse tumor tissues after SH2B3 knockdown/overexpression. (n = 6, mean ± SD, **P < 0.01).

**Figure 4:**
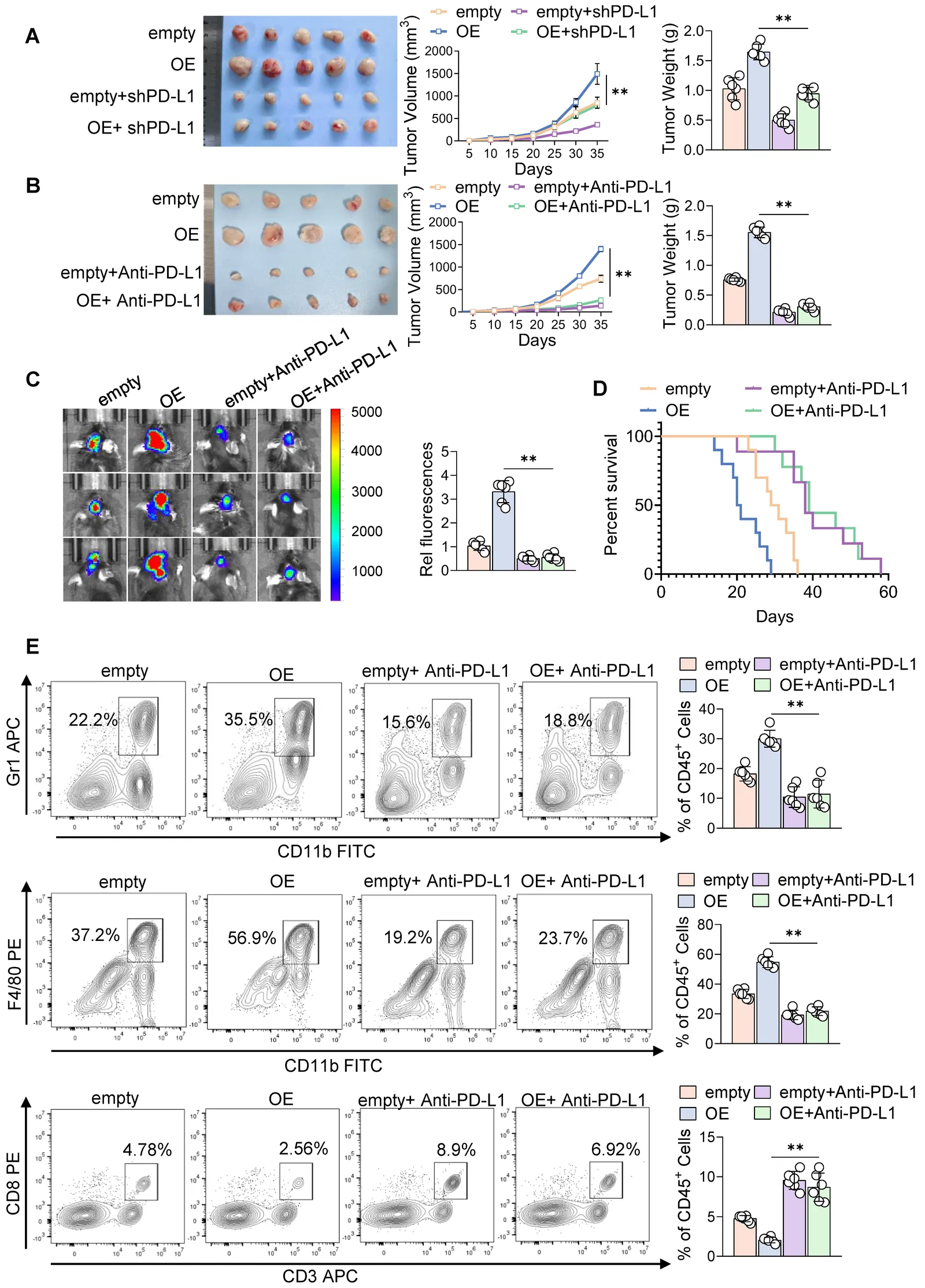
** SH2B3 Drives Glioblastoma Progression *In Vivo* by Enhancing PD-L1-Mediated Immune Evasion. A** Empty, OE-SH2B3, empty+shPD-L1 and OE-SH2B3+shPD-L1 GL261 cells were injected subcutaneously into the flank of C57BL/6 mice to observe tumor proliferation ability. Representative images of excised tumors are shown. Tumor volume was measured (n = 6, mean ± SD, **P < 0.01). Tumor weight was assessed (n = 6, mean ± SD, **P < 0.01). **B** Empty and OE GL261 cells were injected subcutaneously into the flank of C57BL/6 mice, followed by treatment with or without anti-PD-L1 monoclonal antibody (mAb) to evaluate the effect of PD-L1 blockade on SH2B3-overexpression-induced tumor growth. Representative images of excised tumors are shown. Tumor volume was measured (n = 6, mean ± SD, **P < 0.01). Tumor weight was assessed (n = 6, mean ± SD, **P < 0.01). **C** Mice were implanted intracranially with vector-control or SH2B3-overexpressing GL261 cells and subsequently treated with isotype control or anti-PD-L1 antibody into C57BL/6 mice to evaluate glioblastoma growth *in vivo*. Representative *in vivo* bioluminescence imaging images are shown. Relative bioluminescence intensity was quantified (n = 6, mean ± SD, **P < 0.01). **D** Kaplan-Meier survival analysis was performed in intracranial glioma-bearing mice (n = 10, **P < 0.01, Log-rank test).** E** Flow cytometry was performed to analyze immune cell populations in intracranial tumor tissues from mice. The proportions of CD11b⁺Gr-1⁺ myeloid-derived suppressor cells (MDSCs), CD11b⁺F4/80⁺ macrophages, and CD8⁺ T cells among CD45⁺ immune cells were quantified. Representative flow cytometry plots and quantitative statistics are shown (n = 6, mean ± SD, **P < 0.01).

**Figure 5:**
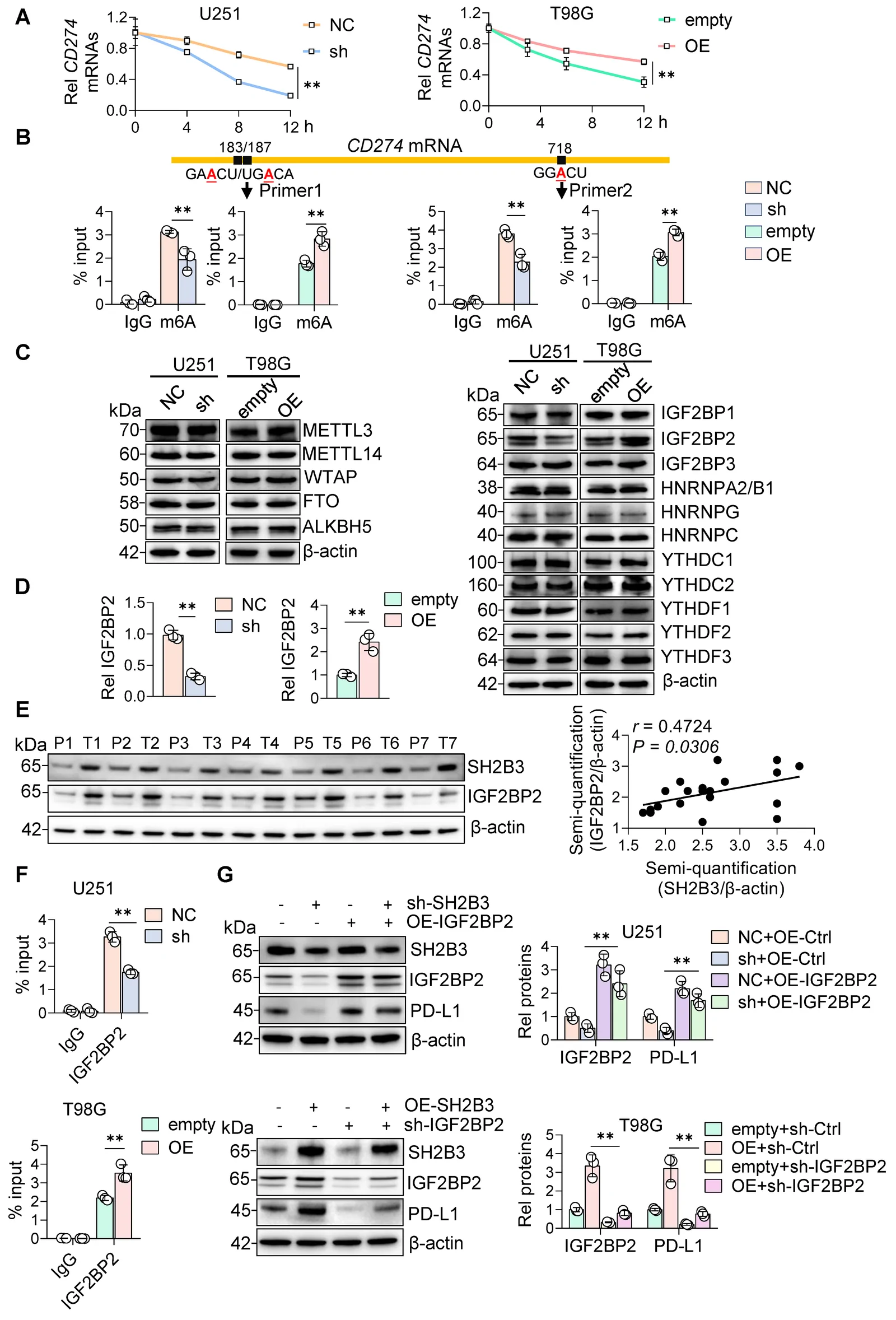
** SH2B3 enhances PD-L1 mRNA stability via IGF2BP2. A** The mRNA stability of PD-L1 was detected after ActD treatment in U251 cells with SH2B3 knockdown and in T98G cells with SH2B3 overexpression (n = 3, mean ± SD, **P < 0.01). **B** Schematic illustration of the putative m⁶A-enriched regions within PD-L1 mRNA and MeRIP-qPCR analysis of PD-L1 transcript enrichment in the m⁶A-IP fraction in U251 cells with SH2B3 knockdown and in T98G cells with SH2B3 overexpression (n = 3 biological replicates, mean ± SD, Two-way ANOVA with Tukey's post hoc test, **P < 0.01). **C** Western blotting detected the expression of different m⁶A methylation-related enzymes expression in U251 cells with SH2B3 knockdown and in T98G cells with SH2B3 overexpression. **D** Densitometric quantification of IGF2BP2 protein levels shown in panel C (n = 3, mean ± SD, **P < 0.01). **E** Western blotting was performed to detect SH2B3 and IGF2BP2 protein levels in paired glioma tissues (T) and peritumoral tissue (P), followed by correlation analysis of semiquantified protein levels (Pearson correlation coefficient *r* = 0.4724, *P* = 0.0306). **F** RIP-qPCR using an anti-IGF2BP2 antibody was performed to assess PD-L1 mRNA enrichment in U251 cells with SH2B3 knockdown and in T98G cells with SH2B3 overexpression (n = 3, mean ± SD, **P < 0.01). **G** Western blotting was performed to detect SH2B3, IGF2BP2, and PD-L1 expression in U251 cells with SH2B3 knockdown and in T98G cells with SH2B3 overexpression, followed by densitometric quantification (n = 3, mean ± SD, **P < 0.01).

**Figure 6:**
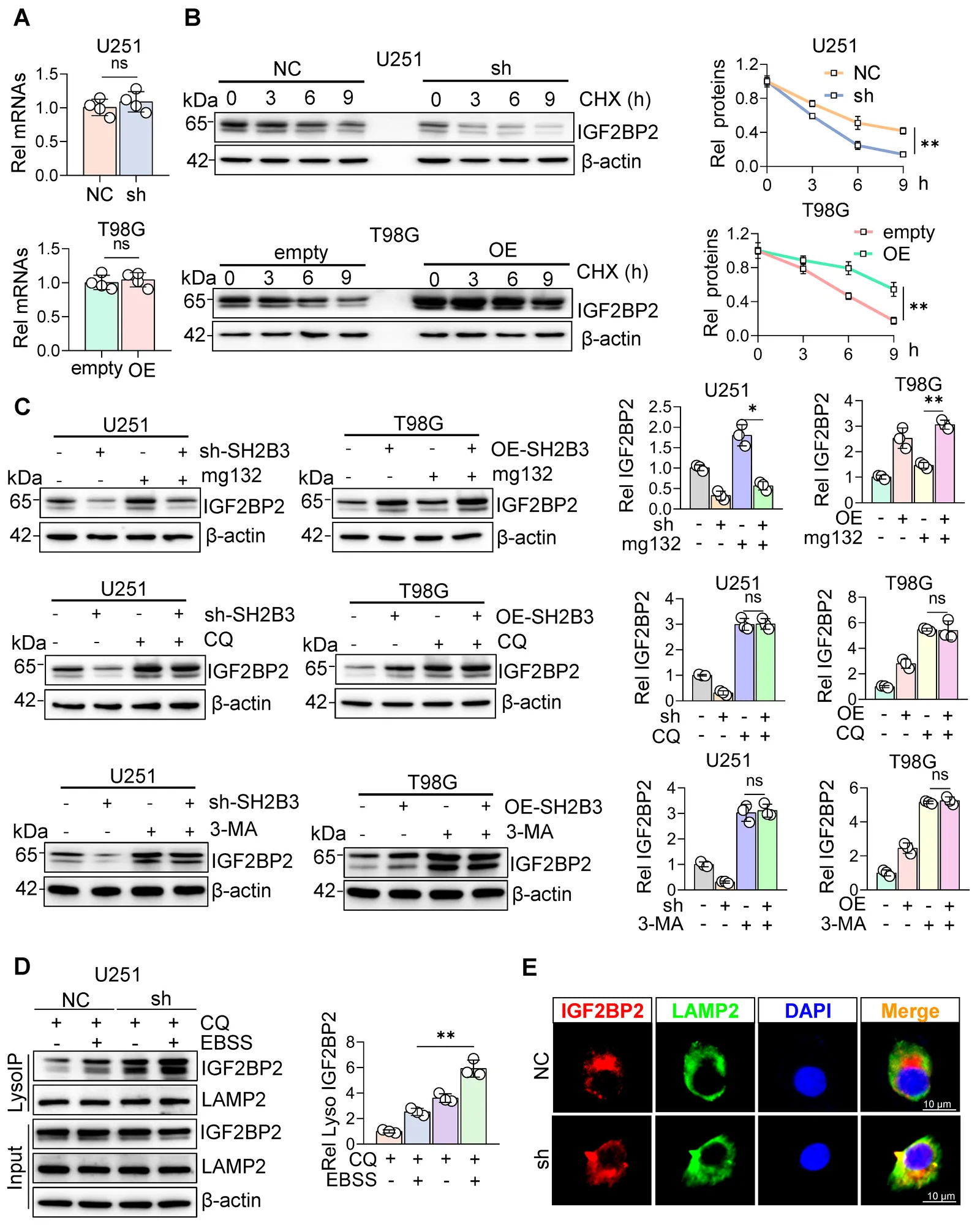
** SH2B3 prevents autophagy-lysosomal degradation of IGF2BP2. A** RT-qPCR analysis of IGF2BP2 mRNA levels in U251 cells with SH2B3 knockdown and in T98G cells with SH2B3 overexpression (n = 4, mean ± SD, ns, nonsignificant). **B** CHX chase assays were performed to assess IGF2BP2 protein stability in U251 cells with SH2B3 knockdown and in T98G cells with SH2B3 overexpression, followed by densitometric quantification (n = 3, mean ± SD, **P < 0.01, Two-way ANOVA). **C** Western blotting of IGF2BP2 protein levels in U251 cells with or without SH2B3 knockdown and in T98G cells with or without SH2B3 overexpression after treatment with MG132, chloroquine (CQ), or 3-methyladenine (3-MA), followed by densitometric quantification (n = 3, mean ± SD, *P < 0.05, **P < 0.01, ns, nonsignificant). **D** Lyso-IP assays were performed in U251 cells to assess lysosomal IGF2BP2 accumulation in control and SH2B3-knockdown cells under CQ treatment with or without EBSS stimulation, followed by quantification of relative lysosomal IGF2BP2 levels. Under CQ treatment alone, there was no significant difference between groups (ns); however, under EBSS+CQ treatment, the levels were significantly increased in SH2B3-knockdown cells (n = 3, mean ± SD, **P < 0.01). **E** Immunofluorescence staining of IGF2BP2 (red), LAMP2 (green), and nuclei (DAPI, blue) in U251 cells after CQ and EBSS treatment.

**Figure 7:**
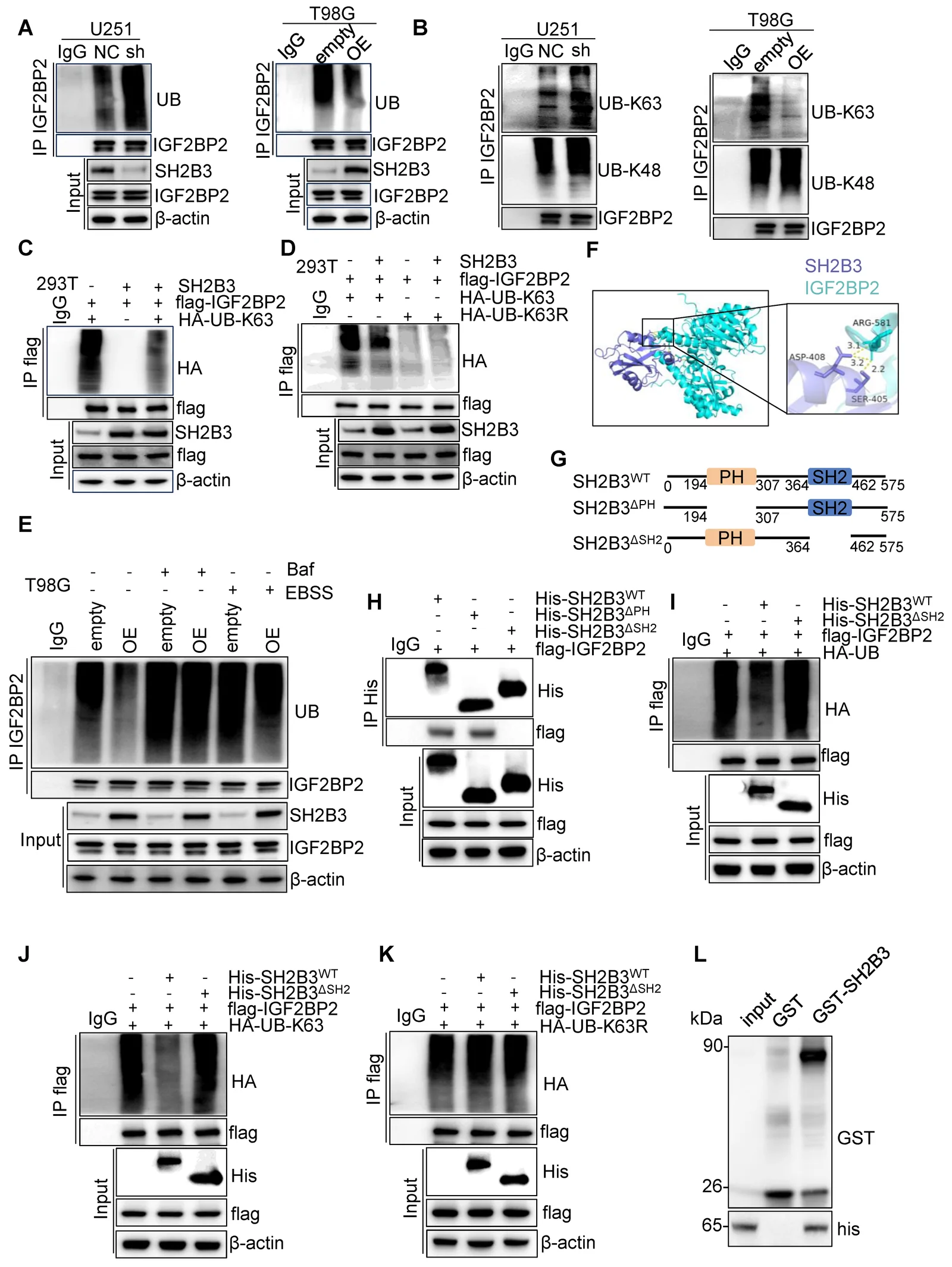
** SH2B3 suppresses K63-linked polyubiquitination of IGF2BP2. A** Ubiquitination analysis of IGF2BP2 in U251 cells with SH2B3 knockdown and in T98G cells with SH2B3 overexpression. Cell lysates were subjected to immunoprecipitation with anti-IGF2BP2 antibody followed by immunoblotting for ubiquitin. **B** Analysis of K63-linked and K48-linked ubiquitination of IGF2BP2 in U251 cells with SH2B3 knockdown and in T98G cells with SH2B3 overexpression. **C** Ubiquitination analysis of IGF2BP2 in HEK293T cells transfected with flag-IGF2BP2 and HA-Ub-K63, with or without SH2B3 overexpression. **D** Ubiquitination analysis of IGF2BP2 in HEK293T cells transfected with flag-IGF2BP2, HA-Ub-K63, or HA-Ub-K63R, in the presence or absence of SH2B3 overexpression. **E** Ubiquitination analysis of IGF2BP2 in T98G cells with or without SH2B3 overexpression after treatment with Bafilomycin A1 (Baf) and/or EBSS. **F** Molecular docking predicts the binding sites of SH2B3 and IGF2BP2.** G** Schematic diagram of full-length SH2B3 and truncation mutants lacking the PH domain (ΔPH) or SH2 domain (ΔSH2). **H** Co-immunoprecipitation analysis in HEK293T cells showing the interaction between flag-IGF2BP2 and His-SH2B3WT, His-SH2B3ΔPH, or His-SH2B3ΔSH2. **I-K** 293T cells were transfected with His-SH2B3^WT^, His-SH2B3^ΔSH2^, flag-IGF2BP2, and HA-Ub (I), HA-Ub-K63 (J), or HA-Ub-K63R (K). Cell lysates were subjected to IP with anti-Flag antibody, followed by immunoblotting with the indicated antibodies. **L** GST pull-down assay using purified recombinant GST-SH2B3 and His-IGF2BP2 proteins showing direct *in vitro* binding (n = 3 biological replicates).

**Figure 8:**
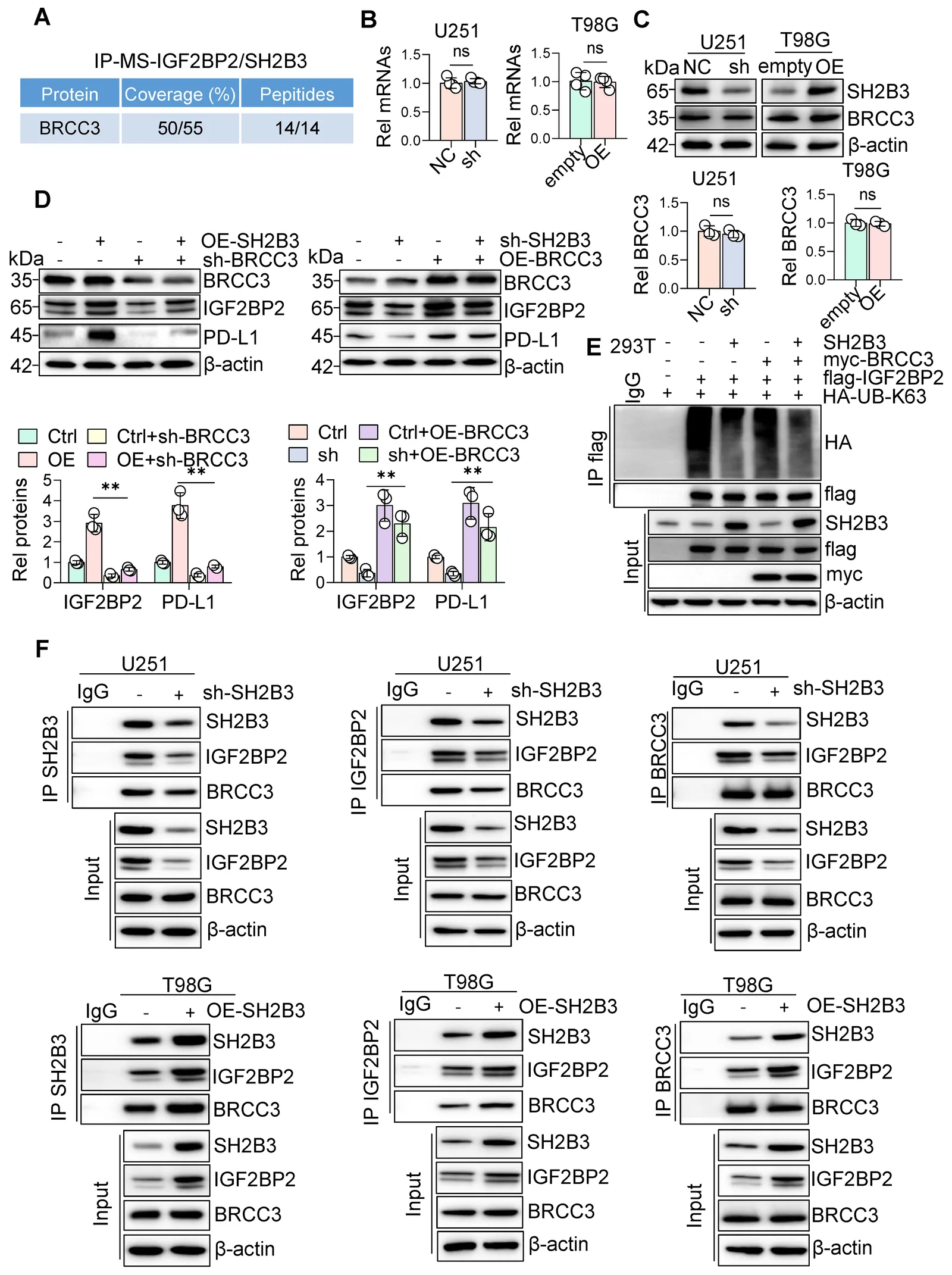
** SH2B3 promotes BRCC3-mediated removal of K63-linked ubiquitin from IGF2BP2. A** IP-MS screening of ubiquitin-related proteins associated with IGF2BP2 and SH2B3. **B** RT-qPCR analysis of BRCC3 mRNA levels in U251 and T98G cells after SH2B3 knockdown or overexpression (n=4, mean ± SD, ns, nonsignificant). **C** Western blotting of BRCC3 protein levels in U251 and T98G cells after SH2B3 knockdown or overexpression (n=3, mean ± SD, ns, nonsignificant). **D** Western blotting of BRCC3, IGF2BP2, and PD-L1 protein levels in U251 and T98G cells following SH2B3 knockdown/overexpression together with BRCC3 overexpression/knockdown, followed by densitometric quantification (n=3, mean ± SD, **P < 0.01). **E** Ubiquitination analysis of IGF2BP2 K63-linked ubiquitination in HEK293T cells transfected with flag-IGF2BP2, HA-Ub-K63, and the indicated combinations of SH2B3 and myc-BRCC3 plasmids (n = 3 biological replicates). **F** Co-immunoprecipitation assays in U251 and T98G cells showing that SH2B3 knockdown weakens, whereas SH2B3 overexpression enhances, the interaction between BRCC3 and IGF2BP2 (n = 3 biological replicates).

**Figure 9:**
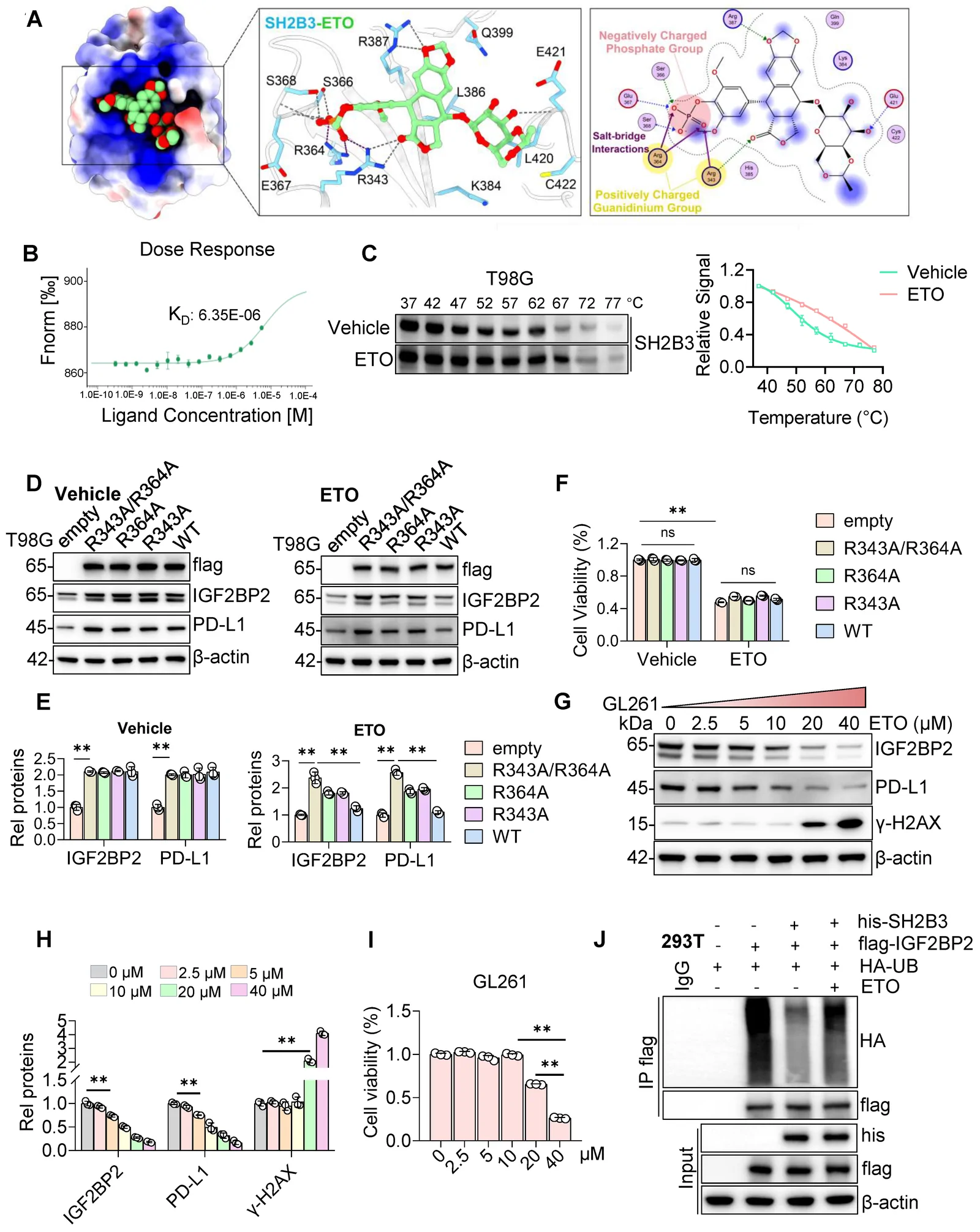
** ETO interferes with the SH2B3-mediated upregulation of IGF2BP2 and PD-L1. A** Molecular docking analysis was performed to predict the binding mode of ETO with SH2B3. **B** Dose-response binding curve showing the interaction between ETO and SH2B3, with an estimated dissociation constant of K_D_ = 6.35 × 10^-6^ M. **C** CETSA was performed to examine the effect of ETO on SH2B3 thermal stability in T98G cells. Relative SH2B3 signal intensity at different temperatures was quantified (n = 3, mean ± SD, Two-way ANOVA, **P < 0.01).** D** Western blotting was performed to detect IGF2BP2 and PD-L1 protein levels in T98G cells expressing empty vector, wild-type SH2B3, or SH2B3 mutants R343A, R364A and R343A/R364A under vehicle or ETO treatment. **E** IGF2BP2 and PD-L1 protein levels shown in panel D were quantified (n = 3, mean ± SD, **P < 0.01). **F** Cell viability was assessed in T98G cells expressing empty vector, wild-type SH2B3, or SH2B3 mutants after vehicle or ETO treatment (n = 3, mean ± SD, **P < 0.01, ns, nonsignificant). **G** Protein levels of IGF2BP2, γ-H2AX, and PD-L1 in GL261 cells treated with escalating doses of ETO were assessed by Western blotting. **H** IGF2BP2, γ-H2AX and PD-L1 protein levels shown in panel G were quantified (n = 3, mean ± SD, **P < 0.01). **I** Cell viability was assessed in GL261 cells treated with increasing concentrations of ETO (n = 3, mean ± SD, **P < 0.01). **J** Ubiquitination assay showing the level of ubiquitinated flag-IGF2BP2 in HEK293T cells co-transfected with His-SH2B3, flag-IGF2BP2, and HA-Ub, followed by ETO treatment.

**Figure 10:**
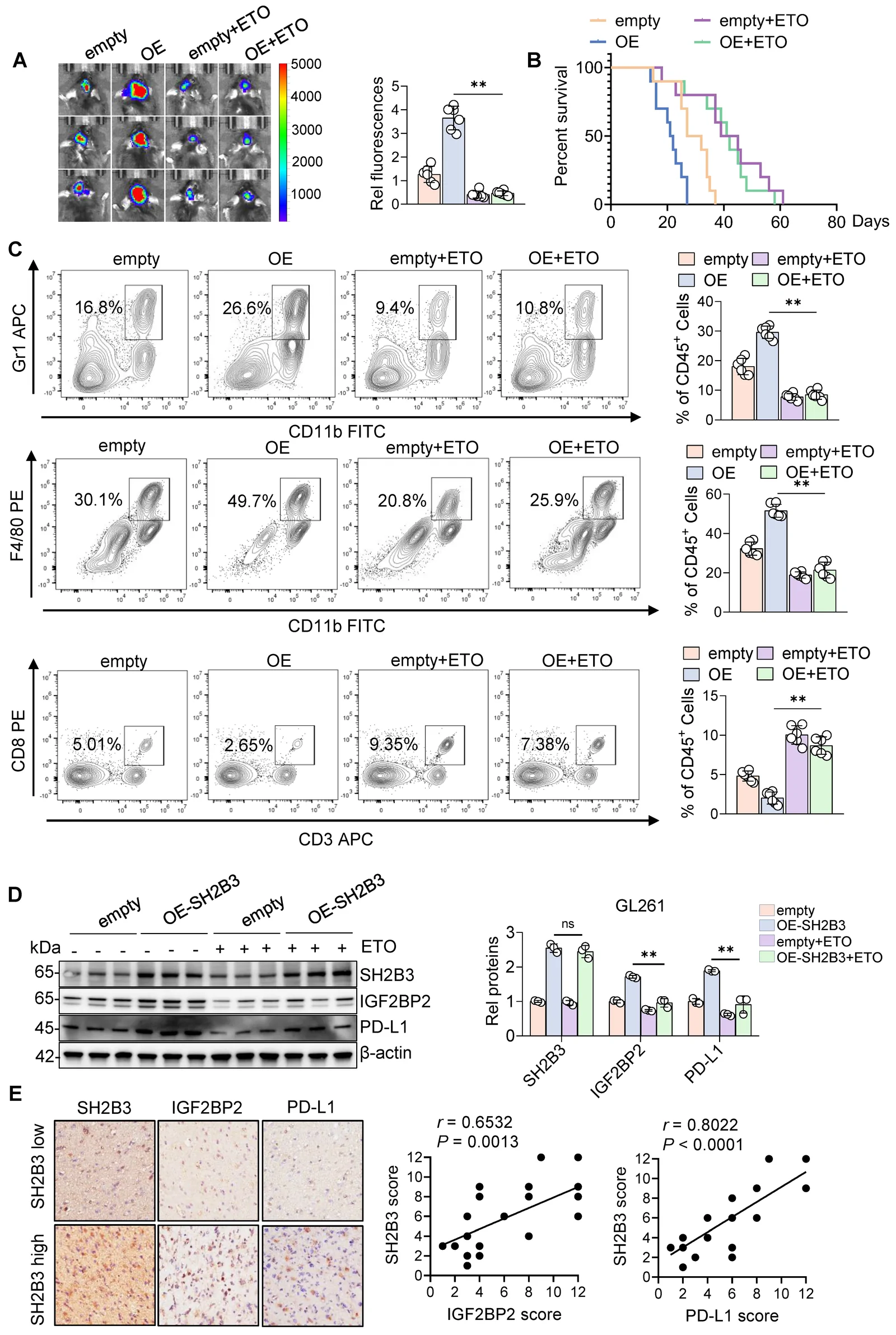
** ETO is associated with reduced tumor burden and altered immune profiles in orthotopic GL261 tumors. A** Empty vector and OE-SH2B3 GL261 cells were intracranially implanted into C57BL/6 mice, followed by vehicle or ETO treatment to evaluate glioblastoma progression *in vivo*. Representative bioluminescence images are shown. Relative bioluminescence signals were quantified (n = 6, mean ± SD, **P < 0.01, One-way ANOVA with Tukey's post hoc test). **B** Kaplan-Meier survival analysis was performed in intracranial glioblastoma -bearing mice implanted with empty vector or OE-SH2B3 GL261 cells and treated with or without ETO (n = 10, **P < 0.01, Log-rank test). **C** Flow cytometry was performed to analyze immune cell populations in intracranial tumor tissues from the indicated groups, and the proportions of CD11b⁺Gr-1⁺ MDSCs, CD8⁺ T cells and CD11b⁺F4/80⁺ macrophages among CD45⁺ cells were quantified (n = 6, mean ± SD, **P < 0.01, One-way ANOVA with Tukey's post hoc test). **D** Western blotting was performed to detect SH2B3, IGF2BP2, and PD-L1 protein levels in intracranial GL261 tumor tissues from the indicated groups. Relative protein levels were quantified (n = 3, mean ± SD, **P < 0.01; ns, not significant, One-way ANOVA with Tukey's post hoc test). **E** Immunohistochemical staining of SH2B3, IGF2BP2, and PD-L1 in human glioma specimens with low or high SH2B3 expression, followed by correlation analysis of SH2B3 with IGF2BP2 and PD-L1 expression (*r* = 0.6532, *P* = 0.0013; *r* = 0.8022, *P* < 0.0001).

**Figure 11:**
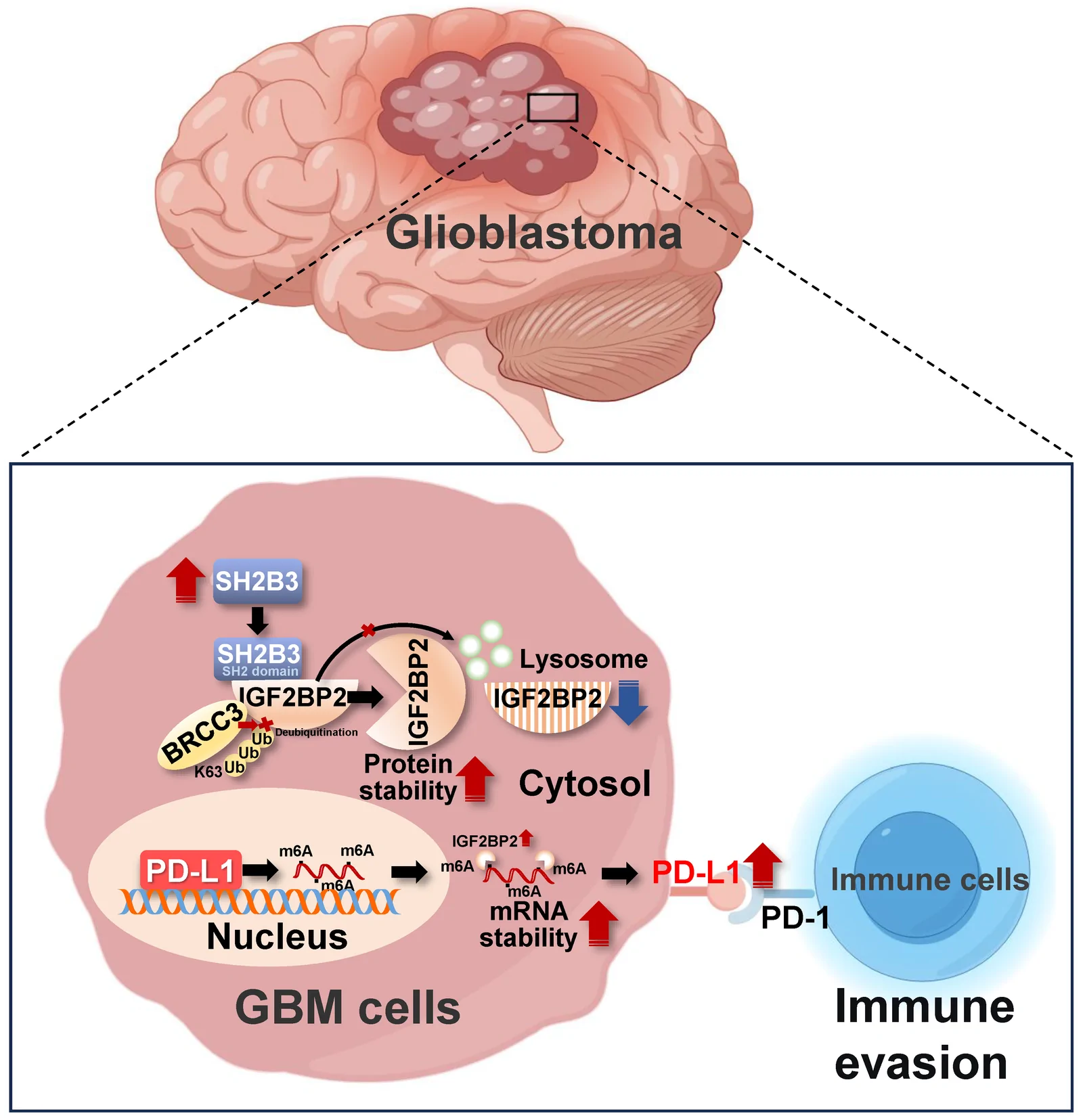
** Schematic working model illustrating the SH2B3/BRCC3/IGF2BP2/PD-L1 axis in GBM.** In the cytosol of GBM cells, elevated SH2B3 promotes the BRCC3-mediated removal of K63-linked polyubiquitin chains from IGF2BP2. This deubiquitination prevents IGF2BP2 from being cleared through the autophagy-lysosome pathway and consequently stabilizes IGF2BP2 protein levels. Stabilized IGF2BP2 acts as an m⁶A reader to recognize and bind m⁶A-modified PD-L1 transcripts, enhancing PD-L1 mRNA stability and subsequently upregulating PD-L1 protein expression. Increased PD-L1 on the tumor cell surface engages PD-1 on immune cells, thereby promoting the formation of an immunosuppressive tumor microenvironment and ultimately facilitating tumor immune evasion.

**Table 1:** Glioma characteristics of patients in SH2B3 low-expression and SH2B3 high-expression groups.

Features	Number	SH2B3 Low	SH2B3 High	*P* value
All patients	39	19	20	
Sex				0.915
Male	27	13	14	
Female	12	6	6	
Age at diagnosis				0.649
<55	15	8	7	
≥55	24	11	13	
Grade				**0.008**
I-II	7	6	1	
III	10	7	3	
IV	22	6	16	

## Data Availability

The raw data supporting the findings of this study are available from the corresponding author upon reasonable request.
