# Supplementary material for: SH2B3 Promotes PD-L1-Mediated Immune Evasion in Glioblastoma via BRCC3-Dependent IGF2BP2 Stabilization
Source: Int J Biol Sci. 2026 Aug 24;22(14):7557–84. doi: 10.7150/ijbs.134558 (PMC13617652; doi:10.7150/ijbs.134558)
Supplement: Supplementary file 1 [file ijbsv22p7557s1.pdf]

Figure S1

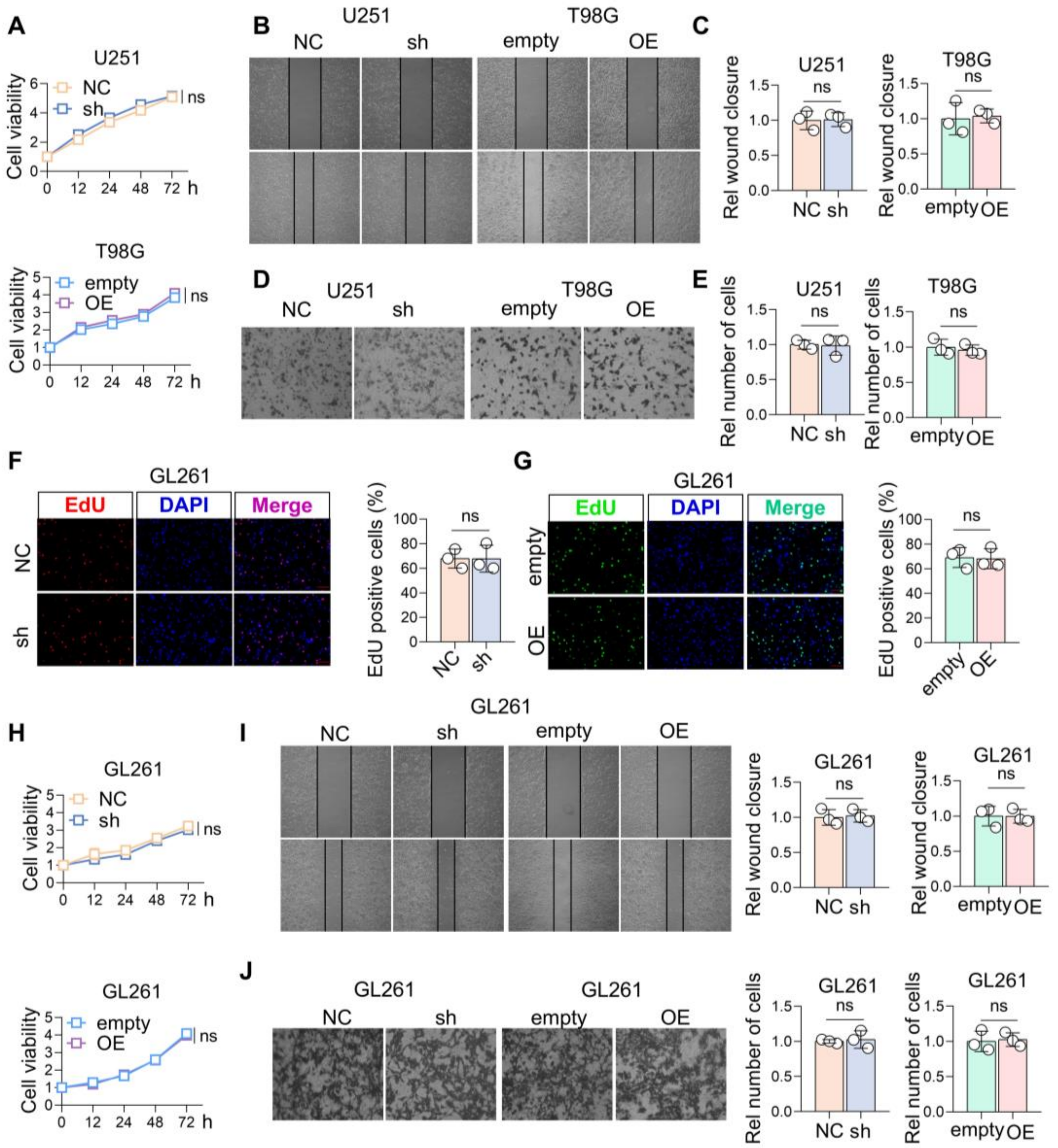

Supplementary Figure 1. SH2B3 does not significantly affect glioblastoma cell proliferation, migration, or invasion in vitro

A Cell viability was assessed by CCK-8 assay in U251 cells transfected with NC or sh-SH2B3 and T98G cells transfected with empty vector or OE-SH2B3 (n = 3, mean ± SD, ns, nonsignificant, Two-way ANOVA). **B** Wound-healing assays were performed in U251 cells transfected with NC or sh-SH2B3 and T98G cells transfected with empty vector or OE-SH2B3, with representative

wound-healing images displayed. **C** Relative wound closure was quantified in U251 cells transfected with NC or sh-SH2B3 and T98G cells transfected with empty vector or OE-SH2B3 (n = 3 independent experiments, mean ± SD, ns, nonsignificant). **D** Transwell invasion assays were performed in U251 cells transfected with NC or sh-SH2B3 and T98G cells transfected with empty vector or OE-SH2B3, with representative images of invaded cells presented. **E** Relative numbers of invaded cells were quantified in U251 cells transfected with NC or sh-SH2B3 and T98G cells transfected with empty vector or OE-SH2B3 (n = 3 independent experiments, mean ± SD, ns, nonsignificant). **F** EdU incorporation assays were performed on GL261 cells transfected with NC or sh-SH2B3; representative EdU, DAPI and merged images are displayed, and EdU-positive cells were quantified (n = 3 independent experiments, mean ± SD, ns, nonsignificant). Scale bar, 100 μm. **G** EdU incorporation assays were performed on GL261 cells transfected with empty vector or OE-SH2B3; representative EdU, DAPI and merged images are displayed, and EdU-positive cells were quantified (n = 3 independent experiments, mean ± SD, ns, nonsignificant). Scale bar, 100 μm. **H** Cell viability was assessed in GL261 cells transfected with NC/sh-SH2B3 or empty vector/OE-SH2B3 (n = 3, mean ± SD, ns, nonsignificant). **I** Wound-healing assays were performed in GL261 cells transfected with NC/sh-SH2B3 or empty vector/OE-SH2B3, and relative wound closure was quantified (n = 3 independent experiments, mean ± SD, ns, nonsignificant,

Two-way ANOVA). **J** Transwell invasion assays were performed in GL261 cells transfected with NC/sh-SH2B3 or empty vector/OE-SH2B3, and relative numbers of invaded cells were quantified (n = 3 independent experiments, mean ± SD, ns, nonsignificant).

Figure S2

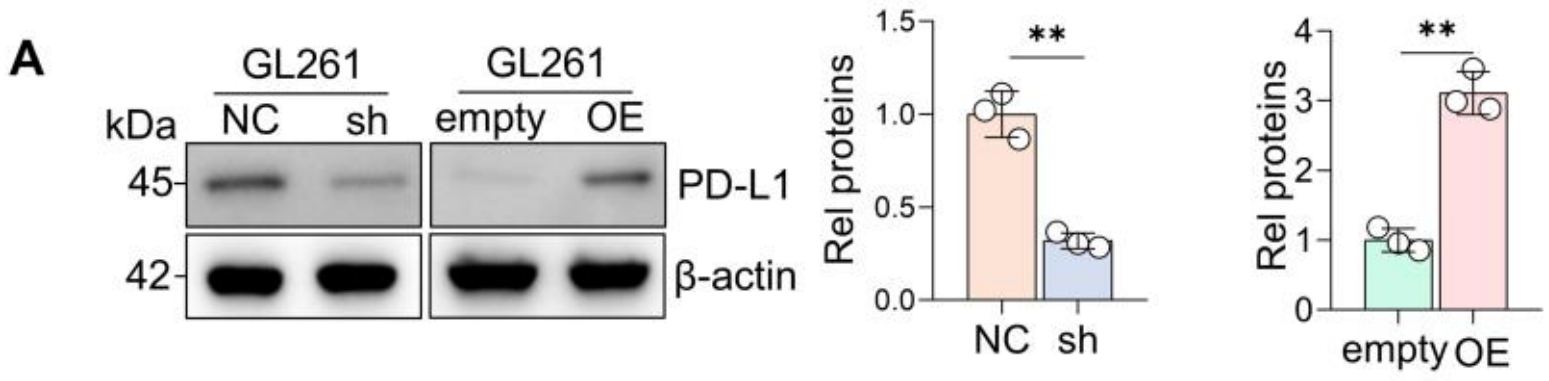

Supplementary Figure 2. SH2B3 regulates PD-L1 expression in GL261 cells

A Western blot analysis was performed to detect PD-L1 protein levels in GL261 cells transfected with NC/sh-SH2B3 or empty vector/OE-SH2B3, followed by quantification of relative PD-L1 protein levels (n = 3, mean ± SD, $**P < 0.01$).

Figure S3

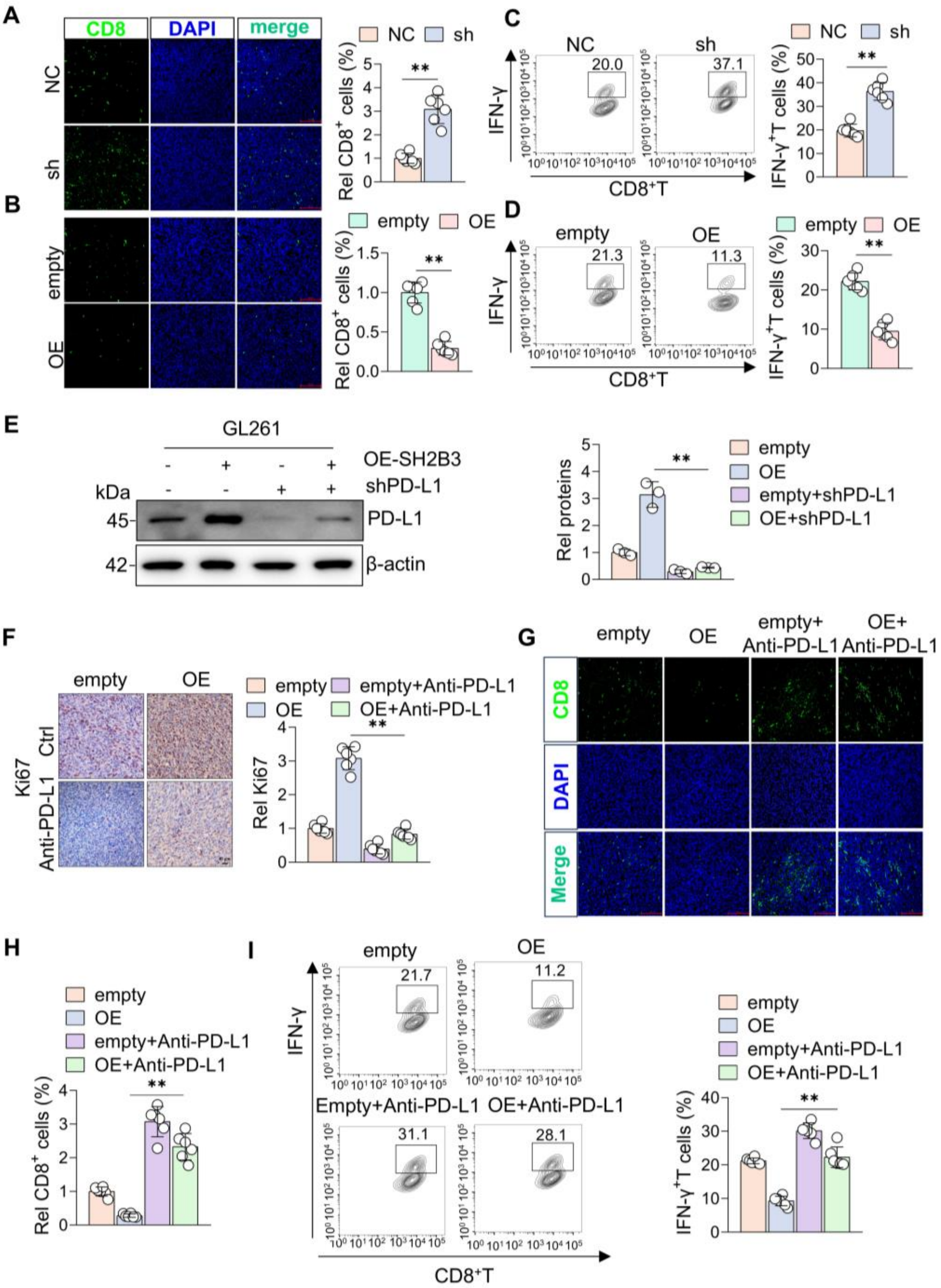

Supplementary Figure 3. SH2B3 suppresses CD8⁺ T-cell infiltration and function in vivo, and PD-L1 blockade reverses these effects

A Immunofluorescence staining was performed to detect CD8⁺ cells in tumor sections of NC and sh-SH2B3 groups, and relative CD8⁺ cell infiltration was

quantified (n = 6, mean ± SD, $^{**}P < 0.01$, unpaired Student's t-test). Scale bar, 100 μm. **B** Immunofluorescence staining was performed to detect $CD8^+$ cells in tumor sections of empty vector and OE-SH2B3 groups, and relative $CD8^+$ cell infiltration was quantified (n = 6, mean ± SD, $^{**}P < 0.01$, unpaired Student's t-test). Scale bar, 100 μm. **C** Flow cytometry was used to analyze IFN-γ^+ $CD8^+$ T cells in tumor tissues from the NC and sh-SH2B3 groups, followed by quantitative analysis of IFN-γ^+ $CD8^+$ T-cell proportions (n = 6, mean ± SD, $^{**}P < 0.01$, unpaired Student's t-test). **D** Flow cytometry was used to analyze IFN-γ^+ $CD8^+$ T cells in tumor tissues from empty vector and OE-SH2B3 groups, followed by quantitative analysis of IFN-γ^+ $CD8^+$ T-cell proportions (n = 6, mean ± SD, $^{**}P < 0.01$, unpaired Student's t-test). **E** Western blot analysis was performed to detect PD-L1 protein levels in GL261 cells transfected with empty vector, OE-SH2B3, empty+sh-PD-L1 or OE-SH2B3+sh-PD-L1, followed by quantification of relative PD-L1 protein levels (n = 3, mean ± SD, $^{**}P < 0.01$, One-way ANOVA with Tukey's post hoc test). **F** Immunohistochemical staining was conducted to detect Ki67 expression in tumor tissues from empty vector, OE-SH2B3, empty+anti-PD-L1 and OE-SH2B3+anti-PD-L1 groups, and relative Ki67 expression levels were quantified (n = 6, mean ± SD, $^{**}P < 0.01$, One-way ANOVA with Tukey's post hoc test). **G** Immunofluorescence staining was performed to visualize $CD8^+$ cells in tumors of the four treatment groups mentioned in panel F; representative CD8, DAPI and merged fluorescence images are presented.

Scale bar, 100 μm. **H** Quantification of relative $CD8^+$ cell infiltration based on immunofluorescence images in panel G (n = 6, mean ± SD, $**P < 0.01$, One-way ANOVA with Tukey's post hoc test). **I** Flow cytometry was performed to detect IFN-γ^+ $CD8^+$ T cells in tumor tissues from empty vector, OE-SH2B3, empty+anti-PD-L1 and OE-SH2B3+anti-PD-L1 groups, and the percentage of IFN-γ^+ $CD8^+$ T cells was quantified (n = 6, mean ± SD, $**P < 0.01$, One-way ANOVA with Tukey's post hoc test).

Figure S4

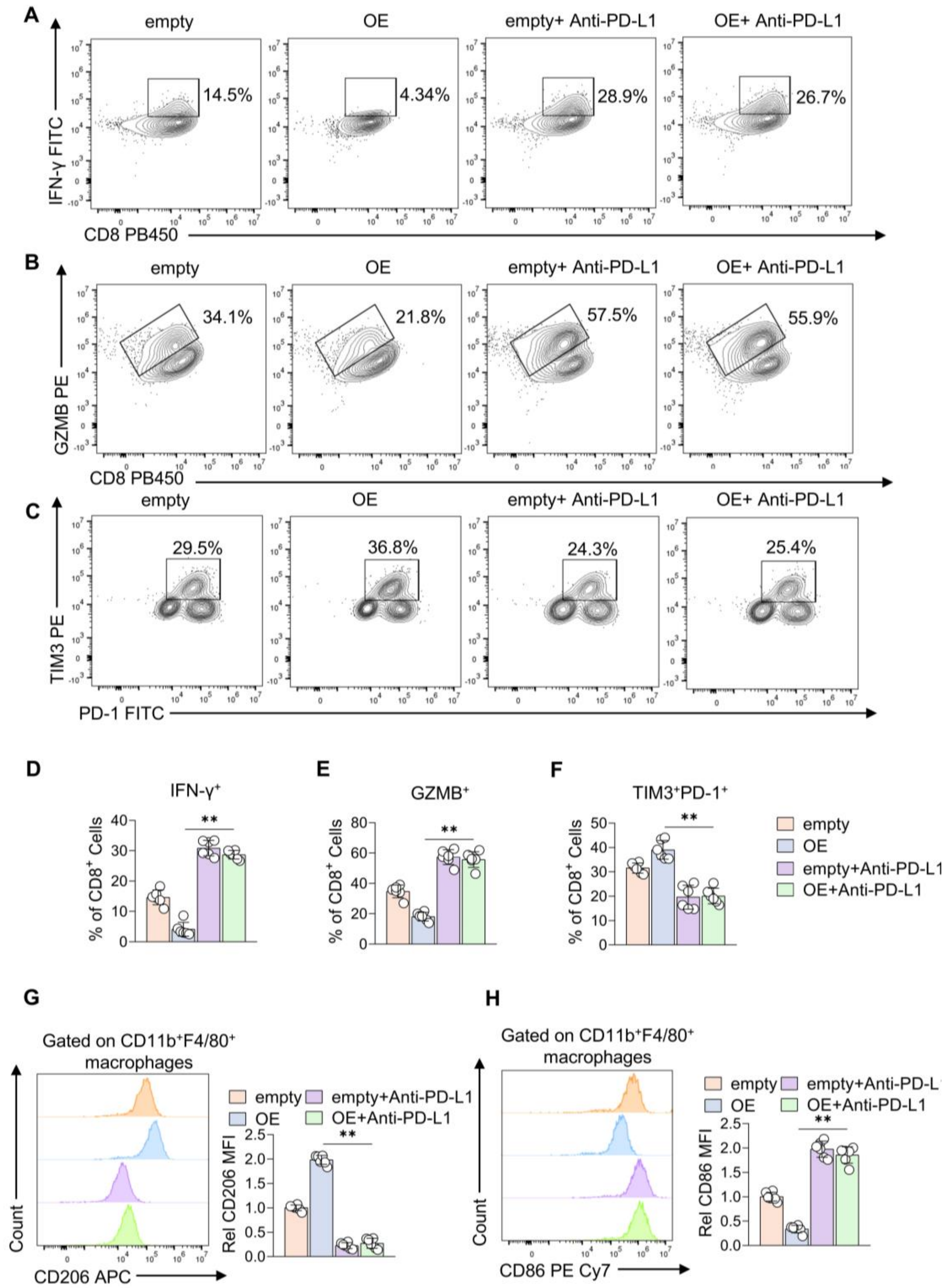

Supplementary Figure 4. Anti-PD-L1 blockade restores CD8⁺ T-cell antitumor activity and alters macrophage polarization in SH2B3-overexpressing tumors

A-C Flow cytometry was performed to analyze IFN-γ$^+$CD8$^+$ T cells, GZMB$^+$CD8$^+$ T cells, and TIM3$^+$PD-1$^+$ exhausted CD8$^+$ T cells, respectively, in tumor tissues from the empty, OE, empty+Anti-PD-L1, and OE+Anti-PD-L1 groups. Representative flow cytometry plots are shown for each subset. **D-F** Quantification of the proportions of IFN-γ$^+$CD8$^+$ T cells, GZMB$^+$CD8$^+$ T cells, and TIM3$^+$PD-1$^+$ exhausted CD8$^+$ T cells corresponding to panels A-C (n = 6, mean ± SD, $^{**}P < 0.01$, One-way ANOVA with Tukey's post hoc test). **G-H** Flow cytometry was performed to assess CD206 and CD86 expression on macrophage populations from the indicated groups, followed by quantification of CD206 and CD86 relative mean fluorescence intensity (MFI), respectively (n = 6, mean ± SD, $^{**}P < 0.01$, One-way ANOVA with Tukey's post hoc test).

Figure S5

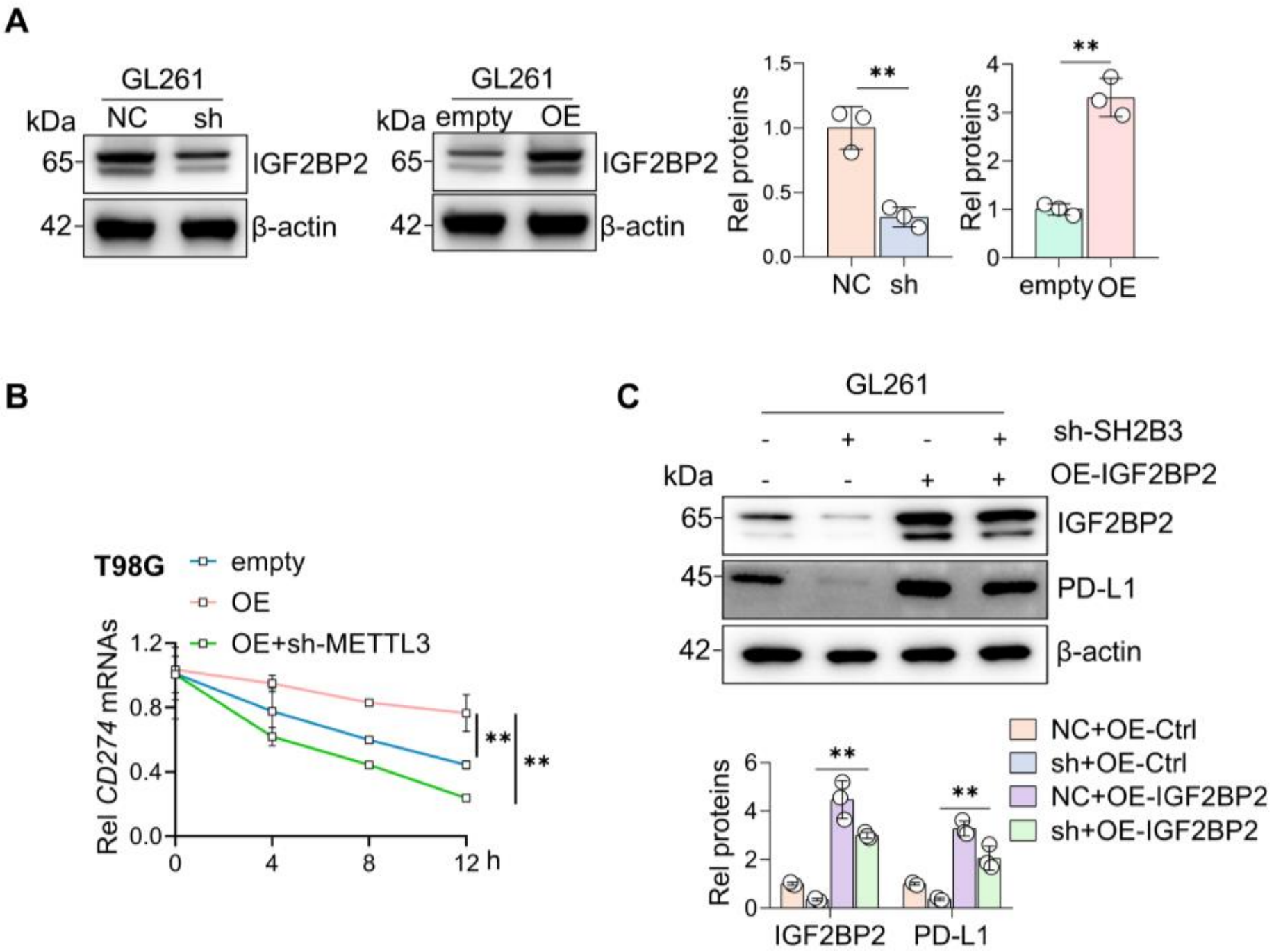

Supplementary Figure 5. SH2B3 Enhances PD-L1 mRNA Stability via IGF2BP2

A Western blot analysis was performed to detect IGF2BP2 protein levels in GL261 cells transfected with NC/sh-SH2B3 or empty vector/OE-SH2B3, followed by quantification of relative IGF2BP2 protein abundance (n = 3, mean ± SD, $^{**}P < 0.01$). **B** PD-L1 mRNA stability was evaluated after transcriptional inhibition in T98G cells transfected with empty vector, OE-SH2B3 or OE-SH2B3+sh-METTL3 (n = 3, mean ± SD, $^{**}P < 0.01$, Two-way ANOVA with Tukey's post hoc test). **C** Western blot analysis was performed to detect IGF2BP2 and PD-L1 protein levels in GL261 cells transfected with NC+OE-Ctrl, sh-SH2B3+OE-Ctrl, NC+OE-IGF2BP2 or sh-SH2B3+OE-IGF2BP2, followed by quantification of relative IGF2BP2 and PD-L1 protein

abundance (n = 3, mean ± SD, $**P < 0.01$, One-way ANOVA with Tukey's post hoc test).

Figure S6

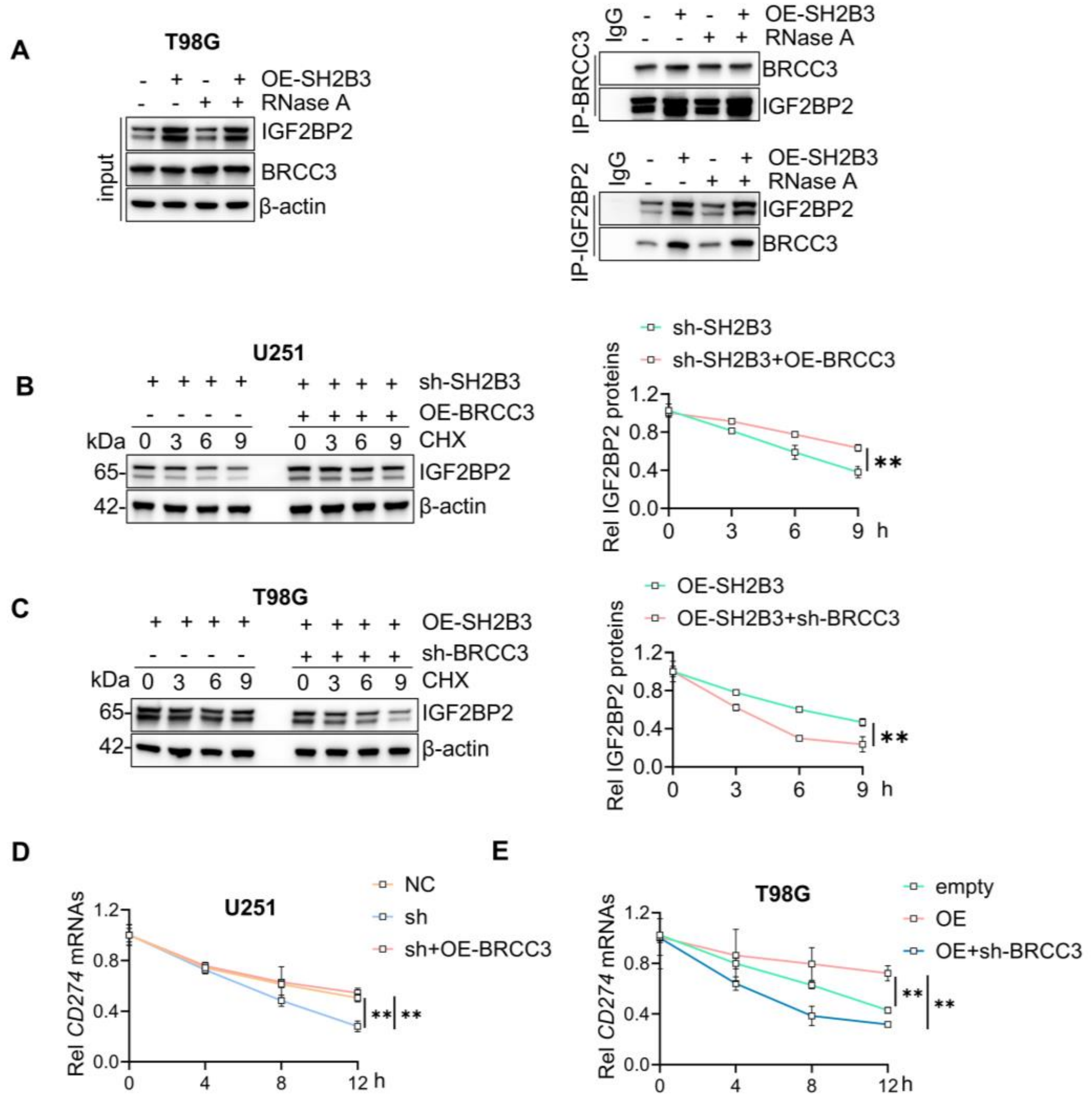

Supplementary Figure 6. BRCC3 Contributes to SH2B3-Mediated Stabilization of IGF2BP2 and PD-L1 mRNA

A Co-immunoprecipitation assays were conducted to detect the interaction between BRCC3 and IGF2BP2 in T98G cells with or without OE-SH2B3 and RNase A treatment; representative immunoblots of input, IP-BRCC3 and IP-IGF2BP2 are displayed (n=3). **B** Cycloheximide chase assays were used to assess IGF2BP2 protein stability in U251 cells transfected with sh-SH2B3 alone or sh-SH2B3 + OE-BRCC3, and relative IGF2BP2 protein abundance

was quantified at designated time points (n = 3, mean ± SD, $^{**}P < 0.01$, Two-way ANOVA). **C** Cycloheximide chase assays were used to assess IGF2BP2 protein stability in T98G cells transfected with OE-SH2B3 alone or OE-SH2B3 + sh-BRCC3, and relative IGF2BP2 protein abundance was quantified at designated time points (n = 3, mean ± SD, $^{**}P < 0.01$, Two-way ANOVA). **D** PD-L1 mRNA stability was detected after transcriptional inhibition in U251 cells transfected with NC, sh-SH2B3 or sh-SH2B3+OE-BRCC3 (n = 3, mean ± SD, $^{**}P < 0.01$, Two-way ANOVA). **E** PD-L1 mRNA stability was measured after transcriptional inhibition in T98G cells transfected with empty vector, OE-SH2B3 or OE-SH2B3+sh-BRCC3 (n = 3, mean ± SD, $^{**}P < 0.01$, Two-way ANOVA).

Figure S7

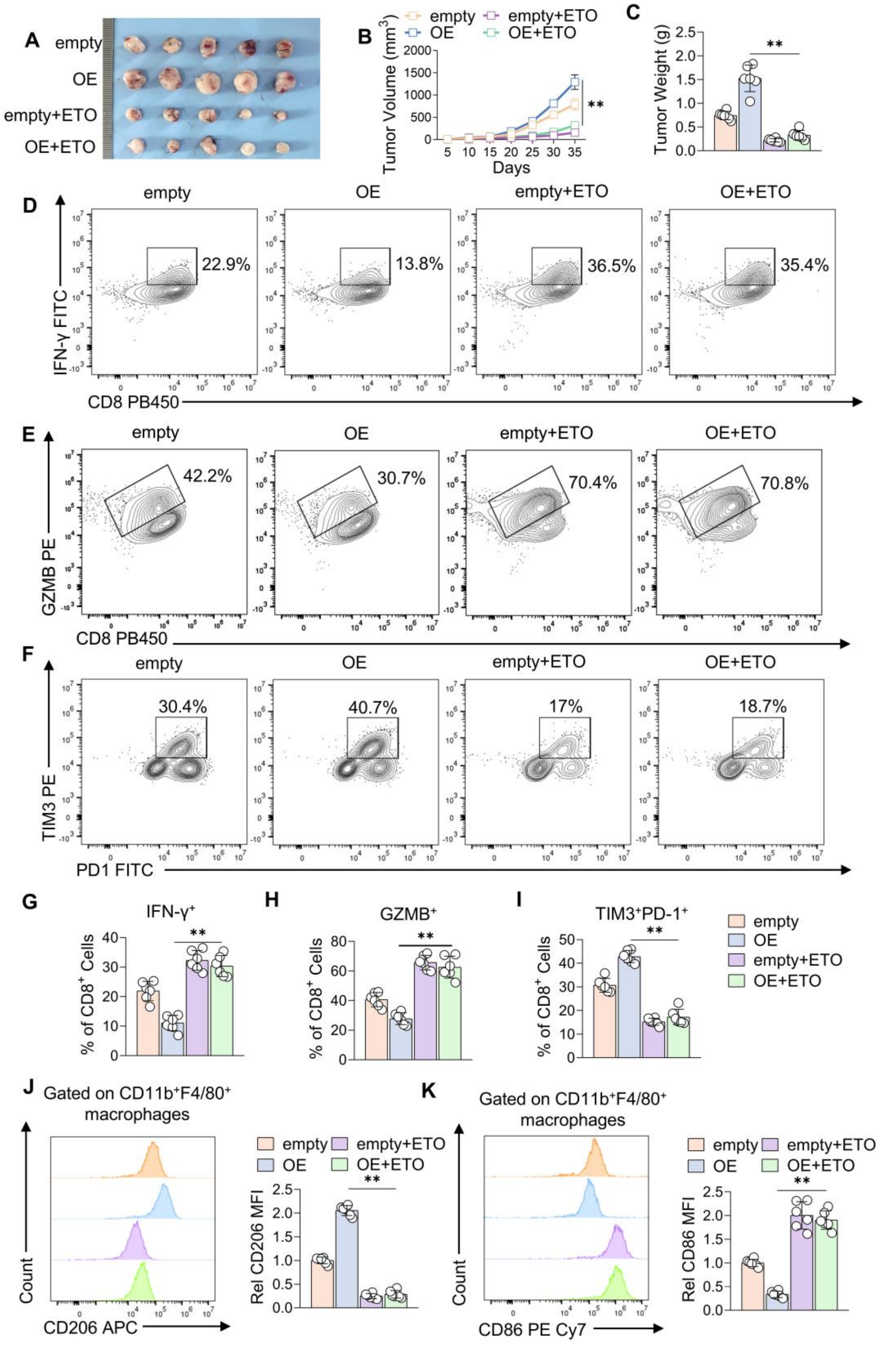

Supplementary Figure 7. ETO restores antitumor immune responses

and suppresses SH2B3-driven glioblastoma growth in vivo

A Empty vector and OE-SH2B3 GL261 cells were subcutaneously injected into the flanks of C57BL/6 mice, which received subsequent vehicle or ETO administration to assess in vivo tumor growth; representative anatomical images of dissected tumors are presented. **B** Tumor volume was quantified for all experimental groups (n = 6, mean ± SD, $^{**}P < 0.01$, Two-way ANOVA). **C** Tumor weight was quantified for all experimental groups (n = 6, mean ± SD, $^{**}P < 0.01$, One-way ANOVA with Tukey's post hoc test). **D-F** Flow cytometry was conducted to detect IFN-γ^+CD8$^+$ T cells, GZMB$^+$CD8$^+$ T cells, and TIM3$^+$PD-1$^+$ exhausted CD8$^+$ T cells in intracranial tumor tissues from empty vector, OE-SH2B3, empty+ETO and OE-SH2B3+ETO groups, with representative flow cytometry plots displayed for each subset. **G-I** Quantification of the proportions of IFN-γ^+CD8$^+$ T cells, GZMB$^+$CD8$^+$ T cells and TIM3$^+$PD-1$^+$ exhausted CD8$^+$ T cells corresponding to panels D-F (n = 6, mean ± SD, $^{**}P < 0.01$, One-way ANOVA with Tukey's post hoc test). **J-K** Flow cytometry was performed to assess CD206 and CD86 expression on macrophage populations from the indicated groups, followed by quantification of mean fluorescence intensity (n = 6, mean ± SD, $^{**}P < 0.01$, One-way ANOVA with Tukey's post hoc test).
